# The power of zero calcium in 82-Rubidium PET irrespective of sex and age

**DOI:** 10.1007/s12350-022-03174-3

**Published:** 2023-01-09

**Authors:** Simon M. Frey, Olivier F. Clerc, Ursina Honegger, Melissa Amrein, Kathrin Thommen, Federico Caobelli, Philip Haaf, Christian E. Müller, Michael J. Zellweger

**Affiliations:** 1 Department of Cardiology, University Hospital Basel, University of Basel, Petersgraben 4, 4031 Basel, Switzerland; 2 Cardiovascular Research Institute Basel (CRIB), University Hospital Basel, University of Basel, Basel, Switzerland; 3 Department of Radiology and Nuclear Medicine, University Hospital Basel, University of Basel, Basel, Switzerland

**Keywords:** ^82^Rubdium positron emission tomography, coronary artery disease, calcium artery calcium score, power of zero, ischemia, risk assessment

## Abstract

**Background:**

Despite clinical suspicion, many non-invasive tests for coronary artery disease (CAD) are normal. Coronary artery calcification score (CACS) is a well-validated method to detect and risk stratify CAD. Patients with zero calcium score (ZCS) rarely have abnormal tests. Therefore, aims were to evaluate CACS as a gatekeeper to further functional downstream testing for CAD and estimate potential radiation and cost savings.

**Methods:**

Consecutive patients with suspected CAD referred for PET were included (n = 2640). Prevalence and test characteristics of ZCS were calculated in different groups. Summed stress score ≥ 4 was considered abnormal and summed difference score ≥ 7 equivalent to ≥ 10% ischemia. To estimate potential radiation/cost reduction, PET scans were hypothetically omitted in ZCS patients.

**Results:**

Mean age was 65 ± 11 years, 46% were female. 21% scans were abnormal and 26% of patients had ZCS. CACS was higher in abnormal PET (median 561 vs 27, *P* < 0.001). Abnormal PET was significantly less frequent in ZCS patients (2.6% vs 27.6%, *P* < 0.001). Sensitivity/negative predictive value (NPV) of ZCS to detect/exclude abnormal PET and ≥ 10% ischemia were 96.8% (95%-CI 95.0%-97.9%)/97.4% (95.9%-98.3%) and 98.9% (96.7%-99.6%)/99.6% (98.7%-99.9%), respectively. Radiation and cost reduction were estimated to be 23% and 22%, respectively.

**Conclusions:**

ZCS is frequent, and most often consistent with normal PET scans. ZCS offers an excellent NPV to exclude an abnormal PET and ≥ 10% ischemia across different gender and age groups. CACS is a suitable gatekeeper before advanced cardiac imaging, and potential radiation/cost savings are substantial. However, further studies including safety endpoints are needed.

**Supplementary Information:**

The online version contains supplementary material available at 10.1007/s12350-022-03174-3.

## Introduction

Myocardial perfusion imaging (MPI) using positron emission tomography (PET) is widely used and well studied for the non-invasive diagnosis of patients with suspected coronary artery disease (CAD).^1,2^ Despite high clinical suspicion, a large proportion of these scans are normal (up to 92%^3^ in certain PET cohorts). Data suggest that over time an increasing number of patients with milder CAD are being sent for testing. Consecutively, low risk tests have increased from around 30% to 80% between 1992 and 2012.^4^ This leads to unnecessary radiation exposure for patients and preventable costs for health care systems. Therefore, it is crucial to optimize allocation of these resources.

Having been introduced more than 30 years ago by Agatston,^5^ the coronary artery calcium score (CACS) has been described as a potential gatekeeper to further CAD testing.^6^ Given its low price, wide availability and its excellent sensitivity and negative predictive value (NPV) to detect and exclude obstructive CAD (sensitivity and NPV ~ 99%), the basic properties of an effective and safe gatekeeper are given.^6-8^

Used in addition to traditional risk factors, CACS can improve risk classification significantly, particularly by reclassification of patients in the intermediate risk group.^9^ Furthermore, its prognostic power in predicting mortality and cardiovascular events is excellent.^6,10-13^ Additionally, the amount of inducible ischemia—a surrogate for obstructive CAD—correlates significantly with coronary artery calcification (CAC).^3,13-18^

Hence, CACS may be an optimal gatekeeper. Especially, patients with zero calcium score (ZCS) are potential candidates to be deferred from further testing since they have an excellent prognosis with an annualized event rate consistently < 1% and an overall mortality of 3% over 15 years.^6,10-13,19,20^

However, most of the data on ischemia and CACS arise from SPECT cohorts.^18^ PET offers more diagnostic accuracy, but only very limited data are available from small PET studies.^3,13-17^ In addition, the diagnostic test characteristics of ZCS have not been sufficiently studied separately in male and female individuals and in different age groups.

Hence, the aims of this study carried out in consecutive patients were twofold: (1) to evaluate the diagnostic value of ZCS to exclude an abnormal perfusion scan or ischemia involving ≥ 10% of the myocardium depending on age and sex; (2) identify and analyze patients who would have been classified false negative if ZCS was used as gatekeeper.

## Methods

### Study design and patient selection

All consecutive patients undergoing a ^82^Rubidium(Rb)-PET scan at the University Hospital Basel from 2016 until end of January 2022 were identified (n = 5151). Data were extracted from the electronic patient record. Patients without known CAD and with complete data on CACS and semi-quantitative analysis of PET perfusion were included for the analysis (n = 2640). Per local protocol, CACS is not performed in patients with known CAD (i.e., post-stenting, bypass) which explains the reduction of sample size by ~ 50%. The study was carried out according to the principles of the Declaration of Helsinki and was approved by the local ethics committee (Ethikkommission der Nordwest- und Zentralschweiz (ethics committee of northwestern and central Switzerland), ID: Req-2022-00393).

### Imaging and stress protocol

Imaging protocols were used as described before.^21^ In short, patients were instructed to withhold caffeine-containing products for 24 hours before the test. For the PET study, a whole-body 3D-PET/CT was used (Biograph mCT, Siemens Healthineers, Erlangen, Germany). A low-dose CT scan was obtained for attenuation correction (increment 0.6 mm, soft-tissue reconstruction kernel, 120 keV, CAREDOSE 4D). Subsequently, a second, ECG-triggered non-enhanced low-dose CT during breath hold was acquired for CACS (120 kV, 25 mAs, rotation time 2.1 s, Matrix 128 × 128, slice thickness 3 mm). Thereafter ^82^Rb was intravenously injected in a weight-adjusted manner for rest and stress images (< 100 kg: 1110 MBq, ≥ 100 kg 1480 MBq). After resting imaging acquisition, patients were pharmacologically stressed with adenosine (140 µg/kg/min for 6 min). If contraindications or personal preferences were present (mostly allergic asthma), regadenoson was used instead (400 µg single-dose). Patients were monitored according to the guidelines.^22^

ECG-gated PET images were recorded for rest and stress over 7 minutes in list mode starting with tracer injection. ECG-gated images were analyzed using QGS-QPS software included in the SyngoVia package (Siemens). CACS values were calculated with the corresponding module within the SyngoVia software.

### Images interpretation

Images were analyzed and interpreted by an experienced board-certified nuclear medicine physician and cardiologist as a joint read reaching consensus. A visual semi-quantitative 17-segment model with a 5-point scale (0: normal tracer uptake, 4: no tracer uptake) was used to calculate summed stress (SSS), rest (SRS) and difference score (SDS = SSS-SRS). A SSS ≥ 4 was considered as threshold for an abnormal PET scan. Derived from the maximal score (17 segments × 4 points = 68), an SDS ≥ 7 was considered to be consistent with ≥ 10% of myocardium ischemic (SDS 6.8 = 10% of maximal score) as described in the current guidelines.^1^

Myocardial blood flow (MBF) was automatically calculated with SyngoVia (Siemens Healthineers, Erlangen, Germany) and approved by the readers. Rest, stress MBF (rMBF, sMBF) and myocardial flow reserve (MFR) were calculated. The arterial input function was derived from the dynamic PET data. A single tissue compartment model was used to calculate myocardial perfusion in mL⋅g^−1^⋅min^−1^.^21,23^ Microvascular dysfunction (MVD) was defined as normal PET (SSS < 4), MFR < 2.0 and sMBF < 2.5.

Based on current clinical measurements, radiation dose of CACS was in average 0.3 mSv and Rb-PET as 2.4 mSv (excluding CACS). For estimation of potential financial savings, 400.- and 2600.- CHF (Swiss Francs) were used for CACS and PET, respectively. To assess the amount of potential radiation/cost saving, only radiation/cost of CACS were used in patients with ZCS and compared to the amount if CACS + PET was performed in the overall cohort.

### Statistical analysis

Normally distributed continuous variables are reported as mean ± standard deviation (SD), and statistical testing was performed with unpaired *t* test or ANOVA. Non-normally distributed continuous variables are reported as median ± interquartile range (IQR) and Mann-Whitney U or Kruskal-Wallis tests were used where appropriate.

Categorical variables are displayed using frequencies and percentages and were compared using the Chi-squared test or Fisher’s exact text where appropriate. A *P* value < 0.05 was considered as statistically significant.

Sensitivity, specificity, positive and negative predictive value (PPV, NPV) and positive and negative likelihood ratio (PLR, NLR) were calculated, and receiver operating characteristic (ROC) analysis was performed to determine the area under the curve (AUC). Since CACS might differ according to subgroup, variable cut-offs might be used individually. Pre-defined CACS values (1, 5, 10, 20, 100) were tested empirically in all subgroups.

To estimate the value of CACS and CACS > 0 as a predictor for abnormal PET and ≥ 10% ischemia, a multivariable binary logistic regression model was used. This model was based on univariable analysis and clinical judgment and included the following variables: age, sex, body mass index, symptoms (angina, dyspnoea) and CAD risk factors (hypertension, family history, diabetes, smoking status, cholesterol) and CACS. A backward selection process with a removal criteria of *P* > 0.1 was used.

To identify cut-off values of CACS above/below which a PET scan is highly likely to be abnormal/normal, we calculated 90th percentile of CACS in patients with SSS < 4 or SDS < 7, and 5th percentile of CACS in patients with SSS ≥ 4 or SDS ≥ 7. Prevalence of abnormal PET and ≥ 10% ischemia were derived for the three areas (≥ 90th, > 5th and < 90th, ≤ 5th percentile). Statistical analyses were performed using SPSS™ (version 27) and RStudio (using R version 4.1.2).

### Patient and public involvement

Patients or the public were not involved in the design, or conduct, or reporting, or dissemination plans of our research.

## Results

### Patient population

A total of 2640 patients were analyzed. The mean age was 65 ± 11 years and 46% were female. Baseline characteristics of the patients are displayed in Table 1. Angina and dyspnoea were present in 39% and 60%, respectively. 558 (21%) scans were abnormal, 262 (10%) showed ≥ 10% ischemia and 73 (2.8%) had MVD. 26% of patients had ZCS.

**Table 1:** Baseline characteristics

Age group	Overall	< 40	40–49	50–59	60–69	70–79	≥ 80	*P* value
n =	2640	45	170	633	842	692	258	
Male gender (%)	1429 (54)	26 (58)	103 (61)	383 (61)	471 (56)	328 (47)	118 (46)	< .01
Age (years)	65 (11)	34 (5)	46 (3)	55 (3)	64 (3)	74 (3)	83 (3)	< .01
BMI (kg⋅m^−2^)	29 (6)	35 (9)	30 (7)	29 (6)	29 (6)	28 (6)	26 (5)	< .01
Hypertension	1779 (67)	18 (40)	89 (52)	382 (60)	570 (68)	509 (74)	211 (82)	< .01
No diabetes (%)	2005 (76)	37 (82)	132 (78)	475 (75)	620 (74)	542 (78)	199 (77)	.4
Hypercholesterolemia (%)	1320 (50)	17 (38)	78 (46)	297 (47)	435 (52)	372 (54)	121 (47)	.03
Family history (%)	610 (23)	18 (40)	54 (32)	200 (32)	200 (4)	105 (15)	33 (13)	< .01
Non-smoker (%)	1311 (50)	13 (29)	72 (42)	285 (45)	406 (48)	374 (54)	161 (62)	< .01
Typical or atypical angina (%)	1018 (39)	17 (38)	76 (45)	263 (42)	332 (39)	248 (36)	82 (32)	.03
Dyspnoea (%)	1575 (60)	25 (56)	93 (55)	342 (54)	488 (58)	455 (66)	172 (67)	< .01

Baseline characteristics of patients studied with 82Rb-PET stratified by age group. Values are displayed as mean (SD) or frequency (percentage). ANOVA and Chi-Square tests were used where appropriate

*BMI*, body mass index

### Distribution of calcium score

The median CACS was 62 (IQR 0-374) and increased significantly with age irrespective of sex (*P* < 0.001), as depicted in Supplemental Table S1. Male patients had significantly higher CACS values than female patients irrespective of age and scan result (*P* < 0.05 in all age groups). Patients with an abnormal PET result had a significantly higher CACS compared to patients with normal scans. CACS was higher in male and older patients irrespective of PET result as shown in Supplemental Table S2.

**Figure 1:**
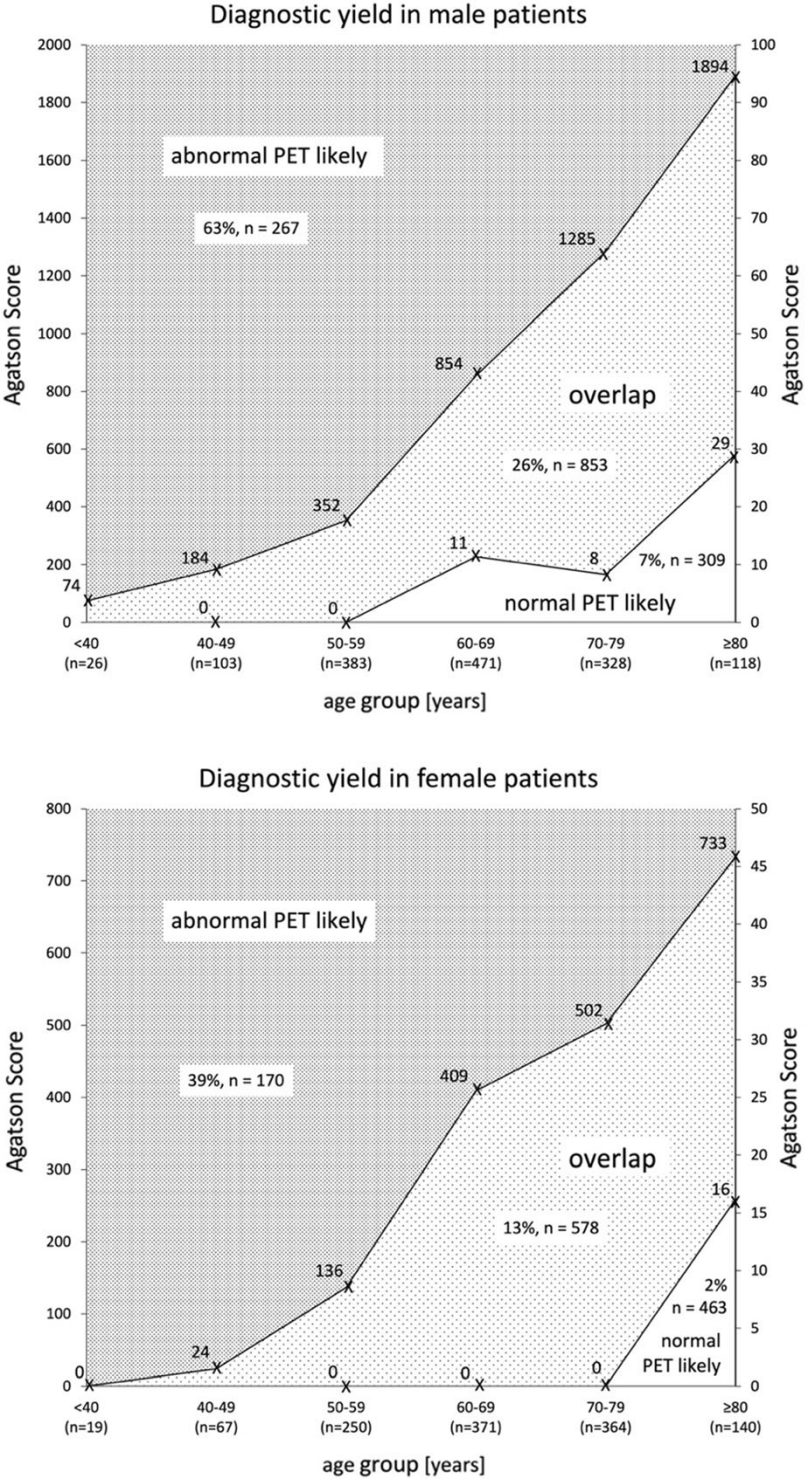
Diagnostic yield of CACS for abnormal scan (SSS ≥ 4). The upper value indicates the 90th percentile of CACS in patients with normal PET (SSS < 4). The lower value indicates the 5th percentile of CACS in patients with abnormal PET (SSS ≥ 4). The prevalence of abnormal PET and number of patients within each area are indicated. For better readability, different scales for absolute CACS were used

**Figure 2:**
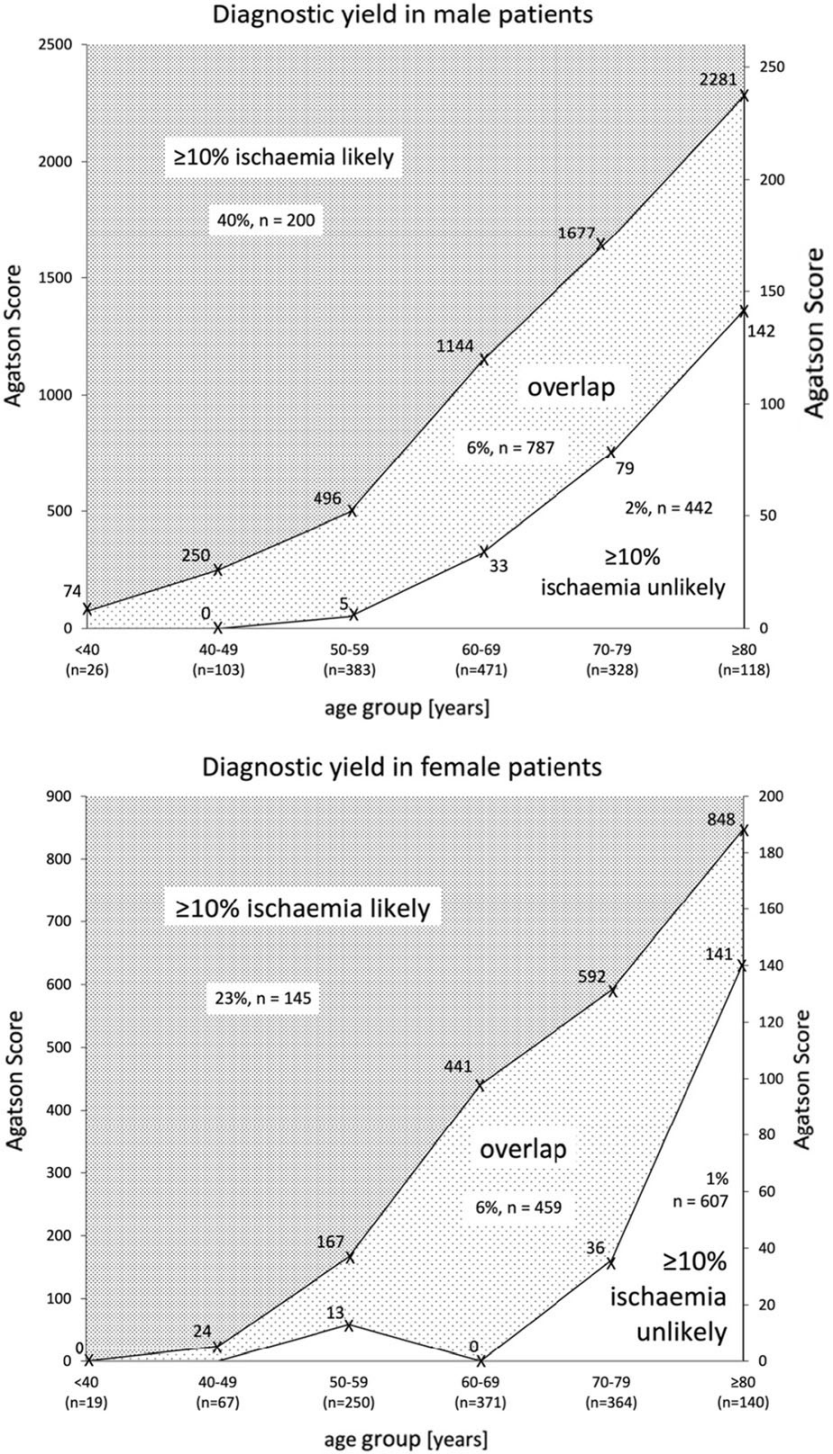
Diagnostic yield of CACS for ≥ 10% ischemia (SDS ≥ 7). The upper value indicates the 90th percentile of CACS in patients with no ischemia involving ≥ 10% of myocardium (SDS < 7). The lower value indicates the 5th percentile of CACS in patients with ≥ 10% ischemia (SDS ≥ 7). The prevalence of ≥ 10% ischemia and number of patients within each area are indicated. For better readability, different scales for absolute CACS were used

Figures 1 and 2 depict the interrelation of CACS percentile, age and sex. Abnormal scans or scans with ≥ 10% ischemia are likely in the dark gray zone (CACS above the 90th percentile of normal scans) and unlikely in the white area (CACS below the 5^th^ percentile of abnormal scans). However, there is considerable overlap.

### Distribution of ZCS

**Figure 3:**
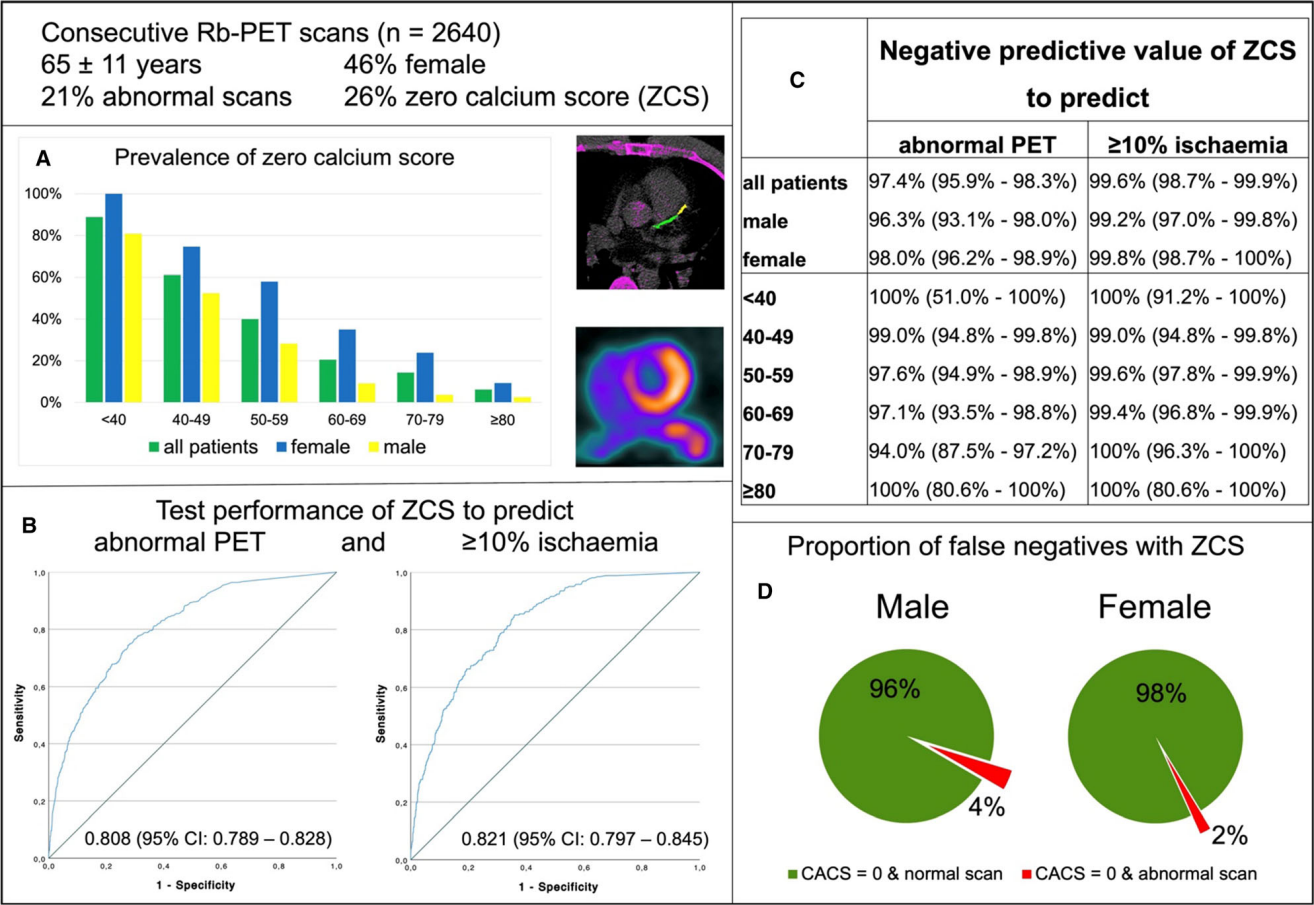
The power of zero calcium score. Zero calcium score (ZCS) is frequent and declines with higher age (A). Calcium score has a good diagnostic performance (ROC analysis for predicting abnormal PET (SSS ≥ 4) and ≥ 10% ischemia (SDS ≥ 7)) (B). Test characteristics of ZCS are excellent to exclude abnormal PET or ≥ 10% ischemia irrespective of age and gender (C). Abnormal PET is infrequent in patients with ZCS (panel D). The illustrating images show a positive Calcium Score in the left main and left anterior descending artery with corresponding anterior/anteroseptal ischemia

ZCS was seen in 685 (26%) patients. Women more frequently had ZCS than men (37% vs 17%, *P* < 0.001). As shown in Figure 3, prevalence of ZCS was highest in young patients < 40 years (100% in women, 81% in men) and declined with higher age to 9% and 3% in women and men ≥ 80 years, respectively (Figure 3, panel A). Overall, the proportion of abnormal scans in patients with ZCS was low (3%) (see also Figure 3, panel D). Correspondingly, the prevalence of an abnormal scan in patients with ZCS was < 5% in the large majority of patients if stratified by gender and age as shown in Table 2. The prevalence of ≥ 10% ischemia among ZCS patients was even lower (0%-2%) as illustrated in Table 2. Of note, no patient > 70 years had ≥ 10% ischemia if CACS was zero.

**Table 2:** Number and percentage of abnormal PET and ≥ 10% ischemia in patients with ZCS and CACS > 0

Age group	Overall			Male			Female		
	ZCS	CACS > 0	*P* value	ZCS	CACS > 0	*P* value	ZCS	CACS > 0	*P* value
*Abnormal PET*									
Overall	18/685 (3%)	540/1955 (28%)	< .001	9/241 (4%)	398/1188 (34%)	< .001	9/444 (2%)	142/767 (19%)	< .001
< 40	0/40 (0%)	1/5 (20%)	.111	0/21 (0%)	1/5 (20%)	.192	0/19 (0%)	0/0 (0%)	ND
40–49	1/104 (1%)	12/66 (18%)	< .001	1/54 (2%)	10/49 (20%)	.003	0/50 (0%)	2/17 (12%)	.062
50–59	6/253 (2%)	85/380 (22%)	< .001	5/108 (5%)	72/275 (26%)	< .001	1/145 (1%)	13/105 (12%)	< .001
60–69	5/173 (3%)	176/669 (26%)	< .001	1/43 (2%)	139/428 (33%)	< .001	4/130 (3%)	37/241 (15%)	< .001
70–79	6/99 (6%)	169/593 (29%)	< .001	2/12 (17%)	119/316 (38%)	.222	4/87 (5%)	50/277 (18%)	.002
≥ 80	0/16 (0%)	97/242 (40%)	< .001	0/3 (0%)	57/115 (50%)	.244	0/13 (0%)	40/127 (32%)	.02
*≥ 10% ischemia*									
Overall	3/685 (0.4%)	259/1955 (13%)	< .001	2/241 (0.8%)	194/1188 (16%)	< .001	1/444 (0.2%)	65/767 (8%)	< .001
< 40	0/40 (0%)	1/5 (20%)	.111	0/21 (0%)	1/5 (20%)	.192	0/19 (0%)	0/0 (0%)	ND
40–49	1/104 (1%)	7/66 (11%)	.006	1/54 (2%)	6/49 (12%)	.051	0/50 (0%)	1/17 (6%)	.254
50–59	1/253 (0.4%)	47/380 (12%)	< .001	1/108 (1%)	40/275 (15%)	< .001	0/145 (0%)	7/105 (7%)	.002
60–69	1/173 (1%)	79/669 (12%)	< .001	0/43 (0%)	63/428 (15%)	.003	1/130 (1%)	16/241 (7%)	.008
70–79	0/99 (0%)	84/593 (14%)	< .001	0/12 (0%)	62/316 (20%)	.133	0/87 (0%)	22/277 (8%)	.003
≥ 80	0/16 (0%)	41/242 (17%)	.083	0/3 (0%)	22/115 (19%)	1	0/13 (0%)	19/127 (15%)	.215

Table illustrates the number and percentage of patients with abnormal PET (SSS ≥ 4) and ≥ 10% ischemia (SDS ≥ 7) stratified according to the presence of coronary artery calcification. Fisher’s exact test was used to calculated difference between groups

### CACS to exclude abnormal PET

ROC analysis showed good diagnostic performance of CACS (Figure 3, panel B, Supplemental Figures S1 + S2). AUC of CACS for abnormal PET was 0.781 (95%-CI 0.755-0.807) in males and 0.812 (95%-CI 0.776-0.848) in females. AUC of CACS for ≥ 10% ischemia was 0.787 (95%-CI 0.754-0.819) and 0.848 (95%-CI 0.808-0.889) in males and females, respectively.

In multivariable logistic regression analysis, CACS > 0 was an independent predictor for abnormal PET and ≥ 10% ischemia (Supplemental Tables S3 and S4).

Overall, ZCS was highly sensitive in excluding abnormal PET (sensitivity 96.8% (95%-CI 95.0%-97.9%), NPV 97.4% (95.9%-98.3%), NLR 0.100 (0.063-0.159)). As depicted in Table 3, the test performed well in all age groups. Sensitivity ranged from 92.3% to 100%, and NPV ranged from 94.0% to 100%. In male patients, sensitivity and NPV were between 90.9% and 100%, and 83.3% and 100%, respectively. In female patients, sensitivity and NPV ranged between 90.2% and 100%, and 95.5% and 100%, respectively.

**Table 3:** Test characteristics of ZCS to exclude an abnormal PET

Group	% ZCS	Sensitivity	Specificity	NPV	PPV	PLR	NLR
Abnormal PET (SSS ≥ 4)							
*Cohort*							
All patients	25.9%	96.8%	32.2%	97.4%	27.7%	1.43	0.10
Male	16.9%	97.8%	22.8%	96.3%	33.5%	1.27	0.10
Female	36.7%	94.0%	41.2%	98.0%	18.6%	1.60	0.15
*Age groups*							
< 40	88.9%	100.0%	90.9%	100.0%	20.0%	11.00	-
40–49	61.2%	92.3%	65.6%	99.0%	18.2%	2.68	0.12
50–59	40.0%	93.4%	45.8%	97.6%	22.4%	1.72	0.14
60–69	20.5%	97.2%	25.6%	97.1%	26.3%	1.31	0.11
70–79	14.3%	96.6%	18.2%	94.0%	28.5%	1.18	0.19
≥ 80	6.2%	100.0%	9.9%	100.0%	40.1%	1.11	-
*Male*							
< 40	80.8%	100.0%	84.0%	100.0%	20.0%	6.25	-
40–49	52.4%	90.9%	57.6%	98.1%	20.4%	2.15	0.16
50–59	28.2%	93.5%	34.0%	95.4%	26.3%	1.42	0.19
60–69	9.1%	99.3%	12.7%	97.7%	32.5%	1.14	0.06
70–79	3.7%	98.3%	4.8%	83.3%	37.7%	1.03	0.34
≥ 80	2.5%	100.0%	4.9%	100.0%	49.6%	1.05	-
*Female*							
< 50	80.2%	100.0%	82.1%	100.0%	11.8%	5.60	-
50–59	58.0%	92.9%	61.0%	99.3%	12.4%	2.38	0.12
60–69	35.0%	90.2%	38.5%	96.9%	15.4%	1.47	0.25
70–79	23.9%	92.6%	27.1%	95.5%	18.1%	1.27	0.27
≥ 80	9.3%	100.0%	13.0%	100.0%	31.5%	1.15	-

Table displays the test characteristics of ZCS for predicting abnormal PET (SSS ≥ 4). %ZCS denotes the proportion of patients with ZCS in each group. Due to low number of cases, the first two age groups were taken together in women

*NPV*, negative predictive values, *PPV*, positive predictive value, *PLR*, positive likelihood ration, *NLR*, negative likely hood ratio, *ZCS*, zero calcium score

Test characteristics for ZCS to detect/exclude ≥ 10% ischemia are summarized in Table 4. Overall, ZCS had an excellent sensitivity (98.9% (95%-CI 96.7%-99.6%)) to detect ≥ 10% ischemia, and NPV was > 98% in all patient’s groups, irrespective of gender and age. Similarly to abnormal PET, sensitivity increased with age.

**Table 4:** Test characteristics of ZCS to exclude ≥ 10% ischemia

Group	% ZCS	Sensitivity	Specificity	NPV	PPV	PLR	NLR
≥ 10% ischemia (SDS ≥ 7)							
*Cohort*							
All patients	25.9%	98.9%	28.8%	99.6%	13.3%	1.39	0.04
Male	16.9%	99.0%	19.5%	99.2%	16.3%	1.23	0.05
Female	36.7%	98.5%	38.9%	99.8%	8.5%	1.61	0.04
*Age groups*							
< 40	88.9%	100.0%	90.9%	100.0%	20.0%	11.00	NA
40–49	61.2%	87.5%	63.6%	99.0%	10.6%	2.40	0.20
50–59	40.0%	97.9%	43.2%	99.6%	12.4%	1.73	0.05
60–69	20.5%	98.8%	22.7%	99.4%	11.8%	1.28	0.06
70–79	14.3%	100.0%	16.4%	100.0%	14.2%	1.20	-
≥ 80	6.2%	100.0%	7.4%	100.0%	16.9%	1.08	-
*Male*							
< 40	80.8%	100.0%	84.0%	100.0%	20.0%	6.25	-
40–49	52.4%	85.7%	55.2%	98.1%	12.2%	1.91	0.26
50–59	28.2%	97.6%	31.6%	99.1%	14.6%	1.43	0.08
60–69	9.1%	100.0%	10.5%	100.0%	14.7%	1.12	-
70–79	3.7%	100.0%	4.5%	100.0%	19.6%	1.05	-
≥ 80	2.5%	100.0%	3.1%	100.0%	19.1%	1.03	-
*Female*							
< 50	80.2%	100.0%	81.2%	100.0%	5.9%	5.31	-
50–59	58.0%	100.0%	59.7%	100.0%	6.7%	2.48	-
60–69	35.0%	94.1%	36.7%	99.2%	6.7%	1.49	0.16
70–79	23.9%	100.0%	25.7%	100.0%	8.0%	1.35	-
≥ 80	9.3%	100.0%	10.7%	100.0%	15.0%	1.12	-

Table displays the test characteristics of ZCS for predicting ≥ 10% ischemia. %ZCS denotes the proportion of patients with ZCS in each group. Due to low number of cases, the first two age groups were taken together in women

*NPV*, negative predictive values, *PPV*, positive predictive value, *PLR*, positive likelihood ration. *NLR*, negative likely hood ratio, *ZCS*, zero calcium score

For both endpoints, sensitivity increased with age. Furthermore, the lower limit of the 95% confidence interval for sensitivity increased with increasing age as shown in Supplemental Tables S5 and S6.

### Performance of different CACS cut-offs (1, 5, 10, 20, 100)

As shown in Supplemental tables S7-S10, sensitivity and NPV decline with increasing cut-off values. CACS ≥ 5 provided a good sensitivity (≥ 95%) to detect an abnormal PET overall (95.5%), in male patients (96.8%), patients ≥ 70 years (96.6%), diabetic patients (97.1%), diabetic males (96.9%) and diabetic females (97.6%) (Supplemental Table S7). CACS ≥ 10 had a similar performance in patients ≥ 80 years (99.0%), male patients ≥ 60 years (95.7%), female patients ≥ 80 (100%) and diabetic females (97.6%) (Supplemental Tables S7-S10). CACS ≥ 20 had a sensitivity ≥ 95% in male patients ≥ 70 years and female patients ≥ 80 years only. CACS ≥ 100 has a sensitivity < 90% in all subgroups.

### Wrongly classified patients/“false negative” PET

Eighteen patients had ZCS and an abnormal PET scan (2.6% of patients with ZCS). Of these, mean age was 64 ± 10 years and 50% were male. Anginal symptoms and dyspnoea were reported in 28% and 61%, respectively. Only one of 18 patients was free from traditional risk factors. Median SRS, SDS and SSS were 2.5 (0.0-5.3), 4.0 (0.0-6.0) and 5.0 (4.0-6.8), respectively. Comparing baseline characteristics and symptoms in ZCS patients between normal and abnormal scans, there was no significant difference except for age (Supplemental Table S11). ZCS patients with an abnormal PET were significantly older (64.2 ± 10.5 vs 57.8 ± 11.5 years, *P* value 0.020). Supplemental Table S12 summarises all patients with abnormal PET and ZCS. In summary, all the above-mentioned patients were < 80 years old and had at least one of the following criteria: symptoms, decreased LVEF, regional wall motion abnormality, or a pre-existing cardiac disease.

Medical records of the above-mentioned patients were screened to check whether the positive PET result had an effect on clinical management. This was the case in four patients (22%) who would have been missed if ZCS was used as a gatekeeper. This accounts for 0.6% of patients with ZCS and 0.15% of the overall cohort only. Table 5 summarizes these four patients. All were symptomatic, had risk factors and three of them had ≥ 10% ischemia.

**Table 5:** Case description of patients with ZCS and abnormal PET that changed clinical decision making

Symptoms	Risk factors	PET result	Clinical course
Male (in his 50 seconds), atypical AP	Severe dyslipidaemia (retrospectively)	No scar, antero-septo-apical ischemia (SDS 6)	Severe stenosis of mid LAD, PCI with 1 × DES
Male (in his 40 seconds ), atypical chest pain, significant risk factors	NIDDM, dyslipidaemia, AHT, former smoker	Non-transmural scar basal inferior (SRS 6) with large ischemia (SDS 9)	2 vessel CAD with CTO of mid RCA and severe stenosis M2. Elective PCI to both lesions
Female (in her 60 seconds), typical AP CCS II, SOB NYHA II, 2 weeks ago arm pain during several hours	Former smoker	Non-transmural scar antero-apical (SRS 3) with ischemia (SDS 7)	No follow-up data available, should have undergone angiogram
Male (in his 50 seconds), SOB NYHA II, LVEF 38% (TTE)	IDDM, dyslipidaemia, former smoker	Large anterior ischemia (SDS 20), predominantly in LAD with corresponding reduced myocardial blood flow during stress	Non-obstructive CAD in angiography, perfusion defect most likely due to microvascular dysfunction compatible with non-ischemic cardiomyopathy

*AP*, angina pectoris; *CAD*, coronary artery disease; *CTO*, chronic total occlusion; *DES*, drug eluting stent; *LAD*, left anterior descending; *NIDDM*, non-insulin dependent diabetes mellitus; *PCI*, percutaneous coronary intervention; *SDS*, summed difference score; *SOB*, shortness of breath; *SRS*, summed rest score

Patients with ZCS had significantly less microvascular dysfunction than patients with CACS ≥ 1 (1.0% and 3.4%, respectively, *P* < 0.001).

### Potential radiation and cost savings

Total calculated radiation dose in all patients was 7128 mSv (2640 patients × 2.7 mSv (CACS 0.3 mSv + PET 2.4 mSv)). If PET was omitted in ZCS patients, total radiation dose would have been 5484 mSv (7128 mSv (total dose) minus 1644 mSv (685 patients × 2.4 mSv)). This results in an overall reduction of 23%. To detect the 18 and 4 patients with abnormal and management changing significant PET finding, the extra radiation dose amounted for 91 and 441 mSv per patient detected, respectively.

Using the same estimation for potential cost savings, total costs of 7,920,000 CHF could be reduced by 1,781,000 CHF, which corresponds to a 22% reduction. To detect the 18 and 4 patients with abnormal and management changing significant PET finding, the costs in this cohort would have amounted for 98,944 CHF and 445,250 CHF per patient detected, respectively.

## Discussion

The main findings of this study are as follows: (1) ZCS has good sensitivity and NPV to diagnose/exclude abnormal PET, and excellent sensitivity and NPV to diagnose/exclude ≥ 10% ischemia in all sex and age groups; (2) if ZCS had been used as a gatekeeper for further (PET-)testing, only a minimal number of PET findings relevant for decision making would have been missed (0.6%, 4/685); and (3) the radiation and costs necessary to detect these potentially missed patients are high.

To the best of our knowledge, this is the first and largest PET-MPI study examining ZCS as a potential gatekeeper in different gender and age groups and evaluating consecutive patients in daily practice.

The current study reveals a strong and independent association between CACS and PET findings. This is in line with previously published studies.^3,13-17^ In the largest one (n = 695), Schenker et al. demonstrated that increasing CACS correlates with ischemic burden.^13^ The group reported a higher proportion of abnormal PET in ZCS patients (16% vs 3%), and consequently a lower NPV to exclude abnormal PET (84% vs 97%). This discrepancy is most likely due to different definitions of abnormal PET (SDS ≥ 2 vs SSS ≥ 4).

In another PET study (n = 206), Esteves et al. examined the absence of CAC to exclude an abnormal PET (SSS > 2). They demonstrated a sensitivity and negative predictive value of 95% and 99%, respectively.^17^

In our study, ZCS performed well to diagnose and exclude abnormal PET and ≥ 10% ischemia.

### CACS to exclude CAD

Despite high sensitivity and negative predictive value, ZCS cannot exclude non-calcified, obstructive plaque.^7,8,12,24,25^ The prevalence of stenoses varies considerably in the literature (1.5%^12^-19%^24^ for ≥ 50% stenosis on computed tomography coronary angiogram (CTCA)).

In a substudy of the CORE64 trial (n = 291), Gottlieb et al. reported the highest proportion of patients with ZCS who had a ≥ 50% luminal stenosis (19%).^24^ Consequently, sensitivity and negative predictive value of ZCS were poor (45%, 68%). However, CTCA tends generally to overestimate the degree of stenosis, and a considerable number of 50% stenoses do not cause ischemia. Additionally, smaller study populations seem to reveal lower NPV as shown in a recent meta-analysis.^26^

Clearly lower proportions of significant stenoses with ZCS were reported from the CONFIRM registry. In a study population of more than 10,000 patients, Villines et al. showed that the majority of patients (96.5%) with ZCS have either no CAD (84%) or non-obstructive disease (13%) on CTCA.^8^ Only 3.5% and 1.4% of patients had stenoses ≥ 50% and ≥ 70%, respectively, and the NPV to exclude stenosis was excellent (96% and 99%, respectively). Even lower values were reported from the PROMISE trial (n = 4209)), in which obstructive CAD was present in only 1.5% of patients with ZCS (and ≥ 70% stenosis in only 0.5%).^12^

Compared to invasive angiogram, ZCS was associated with an extremely low prevalence of obstructive CAD measured with invasive angiogram (< 1%).^7^ The sensitivity to detect ≥ 50% stenoses was 99% in men and 100% in women, respectively. NPV to exclude ≥ 70% stenosis was 99%. ZCS was present in 0.7% of men and 0.02% of women with obstructive CAD.^25^

In our cohort, the prevalence of abnormal PET was 3% despite ZCS, and the prevalence of ≥ 10% ischemia even lower (0.4%) which seems perfectly in line with most of the above-cited findings.

### Can patients with ZCS but abnormal PET be identified?

Despite the high NPV, three percent of patients would have been classified as false negative in our cohort, if ZCS had been used as a gatekeeper for PET. Furthermore, only in 0.6% (4/685), PET findings would have changed decision making. Except for age, there was no other variable that differed between false negative and true negative patients. Usually, cardiologists refer patients to CAD testing based on symptoms, clinical risk factors, pre-test probability of CAD and risk-scores. As shown in this study, with CACS as imaging biomarker, pre-selection could be improved. Nevertheless, few patients would have been misclassified. Descriptive similarities within this heterogeneous group included prevalence of traditional risk factors, symptoms, younger age, echocardiographic abnormalities (e.g., reduced LVEF, regional wall motion abnormalities), arrhythmia and pre-existing cardiac structural problems (previous heart surgery, pacemaker, congenital abnormality). Such findings should be considered as caveats even in patients with ZCS.

The increasing sensitivity and narrowing of the 95% confidence interval with age suggest that ZCS performs better in older patients. Similarly, as derived from the percentiles curves (Figures 1 and 2), it seems that especially males older than 50 years and females older than 70 years are suitable candidates to defer from further testing. This age difference was also described by other groups. Dzaye et al. showed that male patients develop coronary calcification more than 10 years earlier than women.^27^

Nevertheless, more research is needed to identify high-risk patients despite ZCS, especially in the young.

### Subgroup specific cut-offs

Since CACS differs significantly between subgroups, individual cut-offs could be used in certain subgroups. CACS ≥ 5 showed good sensitivity overall, and in particular in male and diabetic patients. CACS ≥ 10 (and eventually CACS ≥ 20) should be used in older age groups only. As discussed above, CACS performs less well in younger patients, for which reason ZCS should be used in this group only.

### Zero calcium score and microvascular dysfunction

Moreover, omitting PET scans in patients with ZCS could potentially miss microvascular dysfunction, which itself is associated with an adverse prognosis.^28^ However, in our cohort, only 7 (1.0%) patients would have been missed. This is lower than reported in other studies.^28^ Differences are likely due to a potential overestimation of MVD if only MFR < 2.0 is used as diagnostic criterion. In this case, patients with high resting MBF classify easily as reduced MFR despite having normal stress perfusion values.

### Calcium score as a gatekeeper

Despite its growing evidence and excellent prognostic power of ZCS, CACS has not been widely used as a gatekeeper in clinical practice. In the CRESCENT trial,^29^ Lubbers et al. randomized symptomatic patients with suspected CAD to CTCA or functional testing. In the CT group, a CACS was performed as a gatekeeper and CTCA was completed only if CACS was 1-400, or if CACS was 0 and the pre-test probability was very high (> 70%). During the short follow-up of 1.2 years, none of the 100 patients with ZCS had an adverse event.

In a slightly different setting, Budoff et al. introduced CTCA with a CACS gatekeeper to the CAD risk stratification algorithm of firefighters and could show a significant reduction in costs (503$ vs 1376$ per person).^30^ However, no data on prognosis are provided from this study.

In an ongoing, large, prospective randomized controlled trial (ACCURATE, NCT03972774), 2500 patients with suspected CAD are being randomized for PET scan versus no further testing in patients with zero calcium. The study aim is to compare cost-effectiveness and safety of the two approaches.

Although obstructive CAD was missed in 1.5% of patients with ZCS in the PROMISE trial, these patients had an even lower event rate compared to patients with a normal functional test (1.4% vs 2.1%).^12^ In our cohort, only 0.4% with ≥ 10% ischemia would have been missed. With these reassuring data, there is enough evidence that CACS should be implemented as a gatekeeper in clinical practice and be validated prospectively in different patient cohorts. This approach seems promising, especially since potential radiation and cost savings would be substantial (22%-23% in our cohort).

In addition, radiation dose could be further improved if CACS scans would be used for attenuation correction. This approach was shown to exhibit excellent correlation with standard attenuation correction scans.^31^ However, the more dose intense, dedicated CACS scan should not be substituted with attenuation scans with the same thresholds since this leads to significant underestimation of coronary calcification, especially in patients with minimal calcification only.^32^

## Limitations

Due to the retrospective study design, there are no outcome data available. However, the pragmatic approach provides real-world data which can be applied in everyday practice. Furthermore, there was no imaging core lab evaluating the CACS or perfusion data. However, the images were analyzed according to current guidelines by a small, steady, and experienced team of Cardiologists and Nuclear Medicine Specialists reaching consensus. Hence, data interpretation was performed in a standardized and homogeneous way.

Abnormal PET findings in the small subgroups of patients < 40 years were rare, resulting in a wide range of confidence intervals (Supplemental Tables S5 + S6). Hence, the high sensitivity to detect abnormal PET or ≥ 10% ischemia of 100% in the youngest age group might be over-estimated.

## Conclusion

CACS is highly efficient to rule out abnormal PET in male and female patients across all age groups. Only 3% of patients would have been wrongly classified if ZCS had been used as a gatekeeper. The potential radiation and cost savings using ZCS as a gatekeeper for ischemia testing would be substantial. Nevertheless, more work is necessary to identify high-risk patients despite ZCS who should not be deferred from further testing.

## New knowledge gained

Our study found that ZCS as a potential gatekeeper to PET MPI exhibits good diagnostic performance to exclude abnormal PET MPI in different age and sex groups. The possible radiation and cost savings are substantial.

## Supplementary Information

Below is the link to the electronic supplementary material.

Supplementary file1 (DOCX 528 kb)

Supplementary file2 (PPTX 1416 kb)

Supplementary file3 (MP3 3072 kb)

## Abbreviations

AUC: Area under the curve
BMI: Body mass index
CAC: Coronary artery calcification
CACS: Coronary artery calcium score
CAD: Coronary artery disease
CI: Confidence interval
CTCA: Computed tomography coronary angiogram
ECG: Electrocardiogram
IQR: Interquartile range
MBF: Myocardial blood flow
MFR: Myocardial flow reserve
MPI: Myocardial perfusion imaging
MVD: Microvascular dysfunction
NIDDM: Non-insulin-dependent diabetes mellitus
NLR: Negative likelihood ratio
NPV: Negative predictive value
IDDM: Insulin-dependent diabetes mellitus
PET: Positron emission tomography
PLR: Positive likelihood ratio
PPV: Positive predictive value
Rb: Rubidium
ROC: Receiver operating characteristic
SPECT: Single photon emission computed tomography
SDS: Summed difference score
SRS: Summed rest score
SSS: Summed stress score
SD: Standard deviation
ZCS: Zero calcium score
