# Supplementary material for: The power of zero calcium in 82-Rubidium PET irrespective of sex and age
Source: J Nucl Cardiol. 2023 Jan 9;30(4):1514–27. doi: 10.1007/s12350-022-03174-3 (PMC10371904; doi:10.1007/s12350-022-03174-3)

# The power of zero calcium in 82-Rubidium PET irrespective of sex and age

Simon M. Frey^a,b^ MD, Olivier F. Clerc^a,b^ MD, Ursina Honegger^b^ MSc, Melissa Amrein^b^ PhD, Kathrin Thommen^a^ MMed MSc, Federico Caobelli^c^ MD, Philip Haaf^a,b^ MD, Christian E. Müller^a,b^ MD, Michael J. Zellweger^a,b^ MD

^a^ Department of Cardiology, University Hospital Basel, University of Basel, Switzerland

^b^ Cardiovascular Research Institute Basel (CRIB), University Hospital Basel, University of Basel, Switzerland

^c^ Department of Radiology and Nuclear Medicine, University Hospital Basel, University of Basel, Switzerland

Address for correspondence:

Simon M. Frey, MD

Department of Cardiology, University Hospital, Petersgraben 4, 4031 Basel, Switzerland

Phone : +41 61 265 44 44 ; Fax : +41 61 265 45 98

Email : simon.frey@usb.ch

# Supplemental material

Supplemental Table S1: Coronary artery calcium score values stratified by age group and sex

|  | age group | overall | <40 | 40-49 | 50-59 | 60-69 | 70-79 | ≥80 | p value |
| --- | --- | --- | --- | --- | --- | --- | --- | --- | --- |
| all patients | median (IQR) | 62 (0 - 374) | 0 (0 - 0) | 0 (0 - 17) | 5 (0 - 97) | 82 (3 - 381) | 176 (20-613) | 330 (65-1052) | p < 0.001 |
|  | Range | 0 - 9167 | 0 - 567 | 0 - 9167 | 0 - 3375 | 0 - 6752 | 0 - 8018 | 0 - 6451 |  |
|  | n = | 2640 | 45 | 170 | 633 | 842 | 692 | 258 |  |
| male | median (IQR) | 131 (7 - 620) | 0 (0 - 0) | 0 (0 - 35) | 36 (0 - 155) | 184 (30 - 608) | 415 (99 - 1218) | 747 (238 - 1725) | p < 0.001 |
|  | Range | 0 - 9167 | 0 - 567 | 0 - 9167 | 0- 2197 | 0 - 6752 | 0 - 8018 | 0 - 6451 |  |
|  | n = | 1429 | 26 | 103 | 383 | 471 | 328 | 118 |  |
| female | median (IQR) | 16 (0 - 180) | 0 (0 - 0) | 0 (0 - 0.5) | 0 (0 - 34) | 13 (0 - 126) | 62 (1- 282) | 202 (39 - 668) | p < 0.001 |
|  | Range | 0 - 3940 | 0 - 0 | 0 - 420 | 0 - 3375 | 0 - 3612 | 0 - 2866 | 0 - 3940 |  |
|  | n = | 1211 | 19 | 67 | 250 | 371 | 364 | 140 |  |

Table illustrating coronary artery calcium score values stratified by age group and sex. Values are displayed as median with interquartile range. The Kruskal-Wallis test was used to compare between age categories. IQR: interquartile range.

Supplemental Table S2: Mean coronary artery (CACS) calcium score depending on scan result, age group and sex

|  | age group | overall | <40 | 40-49 | 50-59 | 60-69 | 70-79 | ≥80 |
| --- | --- | --- | --- | --- | --- | --- | --- | --- |
| all patients | normal (SSS <4) | 27 (0 - 208) | 0 (0 - 0) | 0 (0 - 9) | 2 (0 - 59) | 45 (0 - 221) | 97 (6 - 361) | 199 (44 - 674) |
|  | abnormal (SSS ≥4) | 561 (156 - 1412) | 567* | 32 (12 - 376) | 271 (56 - 788) | 533 (142 - 1339) | 835 (250 - 1690) | 857 (248 - 1924) |
|  | p value | <0.001 | 0.044 | <0.001 | 0.001 | 0.001 | 0.001 | <0.001 |
|  | n = normal + abnormal | 2082 + 558 | 44 + 1 | 157 + 13 | 542 + 91 | 661 + 181 | 517 + 175 | 161 + 97 |
| male | normal (SSS <4) | 63 (2 - 316) | 0 (0 - 0) | 0 (0 - 25) | 7 (0 - 91) | 109 (15 - 354) | 243 (43 - 704) | 494 (108 - 1309) |
|  | abnormal (SSS ≥4) | 670 (192 - 1560) | 567* | 32 (13 - 526) | 207 (58 - 785) | 637 (215 - 1416) | 1111 (320 - 2028) | 1149 (386 - 2267) |
|  | p value | 0.001 | ND | <0.001 | <0.001 | 0.001 | <0.001 | 0.002 |
|  | n = normal + abnormal | 1022 + 407 | 25 + 1 | 92 + 11 | 306 + 77 | 331 + 140 | 207 + 121 | 61 + 57 |
| female | normal (SSS <4) | 6 (0 - 110) | 0 (0 - 0) | 0 (0 - 0) | 0 (0 - 8) | 8 (0 - 98) | 40 (0 - 226) | 80 (10 - 325) |
|  | abnormal (SSS ≥4) | 347 (99 - 927) | * | 84 (10 - ND) | 295 (39 - 800) | 205 (19 - 742) | 428 (106 - 1014) | 634 (212 - 1192) |
|  | p value | 0.001 | ND | 0.038 | <0.001 | <0.001 | <0.001 | <0.001 |
|  | n = normal + abnormal | 1060 + 151 | 19 + 0 | 65 + 2 | 236 + 14 | 330 + 41 | 310 + 54 | 100 + 40 |

The table summarises the median CACS with IQR in patients with normal and abnormal scans. Mann-Whitney U test was used between normal and abnormal scan within each group.

* Since the number of abnormal scans was too low in patients age below 40 (n=1 in men, n=0 in women), no IQR or p-value could be calculated. ND: not determined.

Supplemental Table S3: Multivariable regression analysis predicting abnormal PET (SSS ≥4)

|  | OR (95% CI) | *P*-value |
| --- | --- | --- |
| Age | 1.039 (1.028 – 1.050) | <0.001 |
| Male gender | 2.535 (2.029 – 3.167) | <0.001 |
| CACS > 0 | 7.817 (4.779 – 12.786) | <0.001 |
| Diabetes | 1.367 (1.092 – 1.711) | 0.006 |
| Hypercholesterolaemia | 1.242 (1.012 – 1.524) | 0.038 |
| Smoker (former or current) | 1.139 (1.001 – 1.295) | 0.048 |

Multivariable regression model to predict abnormal PET (SSS ≥4). A backward selection process with a removal criteria of p >0.1 was used. CACS: coronary artery calcium score.

Supplemental Table S4: Multivariable analysis predicting ≥10% ischemia (SDS ≥7)

|  | OR (95% CI) | *P*-value |
| --- | --- | --- |
| Age | 1.031 (1.016 – 1.045) | <0.001 |
| Male gender | 2.375 (1.747 – 3.228) | <0.001 |
| CACS > 0 | 20.177 (6.370 – 63.908) | <0.001 |
| Diabetes | 1.466 (1.099 – 1.954) | 0.009 |
| Hypercholesterolemia | 1.610 (1.221 – 2.124) | <0.001 |
| Angina (typical + atypical) | 1.455 (1.107 – 1.912) | 0.007 |
| Smoker (former or current) | 1.211 (1.021 – 1.437) | 0.028 |

Multivariable regression model to predict ≥10% ischemia (SDS ≥7). A backward selection process with a removal criteria of p >0.1 was used. CACS: coronary artery calcium score.

Supplemental Table S5: Test characteristics of zero calcium score (ZCS) to exclude abnormal PET (SSS ≥4) including 95%-confidence intervals

|  | Prevalence | Sensitivity | Specificity | NPV | PPV | PLR | NLR | n = |
| --- | --- | --- | --- | --- | --- | --- | --- | --- |
| all patients | 25.9% | 0.968 (0.95 - 0.979) | 0.322 (0.302 - 0.342) | 0.974 (0.959 - 0.983) | 0.277 (0.257 - 0.297) | 1.427 (1.38 - 1.475) | 0.100 (0.063 - 0.159) | 2640 |
| male | 16.9% | 0.978 (0.959 - 0.988) | 0.228 (0.203 - 0.255) | 0.963 (0.931 - 0.98) | 0.335 (0.309 - 0.363) | 1.267 (1.221 - 1.314) | 0.097 (0.05 - 0.187) | 1429 |
| female | 36.7% | 0.940 (0.891 - 0.968) | 0.412 (0.383 - 0.442) | 0.980 (0.962 - 0.989) | 0.186 (0.16 - 0.215) | 1.600 (1.5 - 1.707) | 0.145 (0.076 - 0.274) | 1211 |
| Stratified by age groupo | |  |  |  |  |  |  |  |
| <40 | 88.9% | 1.000 (0.207 - 1) | 0.909 (0.788 - 0.964) | 1.000 (0.912 - 1) | 0.200 (0.036 - 0.624) | 11.000 (4.321 - 28.002) | NA | 45 |
| 40-49 | 61.2% | 0.923 (0.667 - 0.986) | 0.656 (0.579 - 0.726) | 0.990 (0.948 - 0.998) | 0.182 (0.107 - 0.291) | 2.684 (2.055 - 3.505) | 0.117 (0.018 - 0.773) | 170 |
| 50-59 | 40.0% | 0.934 (0.864 - 0.969) | 0.458 (0.416 - 0.5) | 0.976 (0.949 - 0.989) | 0.224 (0.185 - 0.269) | 1.722 (1.566 - 1.893) | 0.144 (0.066 - 0.314) | 633 |
| 60-69 | 20.5% | 0.972 (0.937 - 0.988) | 0.256 (0.224 - 0.29) | 0.971 (0.935 - 0.988) | 0.263 (0.231 - 0.298) | 1.306 (1.241 - 1.375) | 0.108 (0.045 - 0.259) | 842 |
| 70-79 | 14.3% | 0.966 (0.927 - 0.984) | 0.182 (0.151 - 0.217) | 0.940 (0.875 - 0.972) | 0.285 (0.251 - 0.323) | 1.180 (1.124 - 1.24) | 0.189 (0.084 - 0.423) | 692 |
| ≥80 | 6.2% | 1.000 (0.962 - 1) | 0.099 (0.062 - 0.155) | 1.000 (0.806 - 1) | 0.401 (0.341 - 0.464) | 1.110 (1.055 - 1.169) | NA | 258 |
| Male patients | |  |  |  |  |  |  |  |
| <40 | 80.8% | 1.000 (0.207 - 1) | 0.840 (0.653 - 0.936) | 1.000 (0.845 - 1) | 0.200 (0.036 - 0.624) | 6.250 (2.546 - 15.344) | NA | 26 |
| 40-49 | 52.4% | 0.909 (0.623 - 0.984) | 0.576 (0.474 - 0.672) | 0.981 (0.902 - 0.997) | 0.204 (0.115 - 0.336) | 2.145 (1.584 - 2.903) | 0.158 (0.024 - 1.031) | 103 |
| 50-59 | 28.2% | 0.935 (0.857 - 0.972) | 0.340 (0.289 - 0.395) | 0.954 (0.897 - 0.98) | 0.263 (0.214 - 0.318) | 1.416 (1.282 - 1.565) | 0.191 (0.081 - 0.452) | 383 |
| 60-69 | 9.1% | 0.993 (0.961 - 0.999) | 0.127 (0.095 - 0.167) | 0.977 (0.879 - 0.996) | 0.325 (0.282 - 0.371) | 1.137 (1.089 - 1.188) | 0.056 (0.008 - 0.405) | 471 |
| 70-79 | 3.7% | 0.983 (0.942 - 0.995) | 0.048 (0.026 - 0.087) | 0.833 (0.552 - 0.953) | 0.377 (0.325 - 0.431) | 1.033 (0.994 - 1.074) | 0.342 (0.076 - 1.536) | 328 |
| ≥80 | 2.5% | 1.000 (0.937 - 1) | 0.049 (0.017 - 0.135) | 1.000 (0.439 - 1) | 0.496 (0.406 - 0.586) | 1.052 (0.993 - 1.113) | NA | 118 |
| Female patients | |  |  |  |  |  |  |  |
| <50 | 80.2% | 1.000 (0.342 - 1) | 0.821 (0.726 - 0.889) | 1.000 (0.947 - 1) | 0.118 (0.033 - 0.343) | 5.600 (3.54 - 8.859) | NA | 86 |
| 50-59 | 58.0% | 0.929 (0.685 - 0.987) | 0.610 (0.547 - 0.67) | 0.993 (0.962 - 0.999) | 0.124 (0.074 - 0.2) | 2.382 (1.92 - 2.956) | 0.117 (0.018 - 0.776) | 250 |
| 60-69 | 35.0% | 0.902 (0.775 - 0.961) | 0.385 (0.334 - 0.438) | 0.969 (0.924 - 0.988) | 0.154 (0.114 - 0.205) | 1.467 (1.286 - 1.674) | 0.254 (0.099 - 0.65) | 371 |
| 70-79 | 23.9% | 0.926 (0.824 - 0.971) | 0.271 (0.225 - 0.323) | 0.955 (0.889 - 0.982) | 0.181 (0.14 - 0.231) | 1.270 (1.148 - 1.406) | 0.273 (0.105 - 0.714) | 364 |
| ≥80 | 9.3% | 1.000 (0.912 - 1) | 0.130 (0.078 - 0.21) | 1.000 (0.772 - 1) | 0.315 (0.241 - 0.4) | 1.149 (1.066 - 1.24) | NA | 140 |

Table displays the test characteristics of ZCS for predicting abnormal PET (SSS ≥4). Prevalence denotes the proportion of patients with ZCS in each group. Due to low number of cases, the first two age groups were taken together in women. NPV: negative predictive values. PPV: positive predictive value. PLR: positive likelihood ration. NLR: negative likely hood ratio. ZCS: zero calcium score.

Supplemental Table S6: Test characteristics of zero calcium score (ZCS) to exclude ≥10% ischemia (SDS ≥7) including 95%-confidence intervals

|  | Prevalence | Sensitivity | Specificity | NPV | PPV | PLR | NLR | n = |
| --- | --- | --- | --- | --- | --- | --- | --- | --- |
| all patients | 25.9% | 0.989 (0.967 - 0.996) | 0.288 (0.27 - 0.307) | 0.996 (0.987 - 0.999) | 0.133 (0.118 - 0.148) | 1.389 (1.349 - 1.429) | 0.040 (0.013 - 0.123) | 2640 |
| male | 16.9% | 0.990 (0.964 - 0.997) | 0.195 (0.174 - 0.218) | 0.992 (0.97 - 0.998) | 0.163 (0.143 - 0.186) | 1.229 (1.192 - 1.268) | 0.052 (0.013 - 0.209) | 1429 |
| female | 36.7% | 0.985 (0.919 - 0.997) | 0.389 (0.361 - 0.417) | 0.998 (0.987 - 1) | 0.085 (0.067 - 0.107) | 1.611 (1.525 - 1.702) | 0.039 (0.006 - 0.273) | 1211 |
| Strafiied by age group |  |  |  |  |  |  |  |  |
| <40 | 88.9% | 1.000 (0.207 - 1) | 0.909 (0.788 - 0.964) | 1.000 (0.912 - 1) | 0.200 (0.036 - 0.624) | 11.000 (4.321 - 28.002) | - | 45 |
| 40-49 | 61.2% | 0.875 (0.529 - 0.978) | 0.636 (0.559 - 0.706) | 0.990 (0.948 - 0.998) | 0.106 (0.052 - 0.203) | 2.403 (1.724 - 3.347) | 0.197 (0.031 - 1.234) | 170 |
| 50-59 | 40.0% | 0.979 (0.891 - 0.996) | 0.432 (0.393 - 0.473) | 0.996 (0.978 - 0.999) | 0.124 (0.095 - 0.161) | 1.725 (1.59 - 1.873) | 0.048 (0.007 - 0.336) | 633 |
| 60-69 | 20.5% | 0.988 (0.933 - 0.998) | 0.227 (0.199 - 0.258) | 0.994 (0.968 - 0.999) | 0.118 (0.096 - 0.145) | 1.278 (1.22 - 1.337) | 0.055 (0.008 - 0.388) | 842 |
| 70-79 | 14.3% | 1.000 (0.956 - 1) | 0.164 (0.137 - 0.196) | 1.000 (0.963 - 1) | 0.142 (0.116 - 0.172) | 1.197 (1.155 - 1.24) | - | 692 |
| ≥80 | 6.2% | 1.000 (0.914 - 1) | 0.074 (0.046 - 0.116) | 1.000 (0.806 - 1) | 0.169 (0.127 - 0.222) | 1.080 (1.04 - 1.121) | - | 258 |
| Male patients |  |  |  |  |  |  |  |  |
| <40 | 80.8% | 1.000 (0.207 - 1) | 0.840 (0.653 - 0.936) | 1.000 (0.845 - 1) | 0.200 (0.036 - 0.624) | 6.250 (2.546 - 15.344) | - | 26 |
| 40-49 | 52.4% | 0.857 (0.487 - 0.974) | 0.552 (0.453 - 0.648) | 0.981 (0.902 - 0.997) | 0.122 (0.057 - 0.242) | 1.914 (1.315 - 2.785) | 0.259 (0.042 - 1.603) | 103 |
| 50-59 | 28.2% | 0.976 (0.874 - 0.996) | 0.316 (0.269 - 0.367) | 0.991 (0.95 - 0.998) | 0.146 (0.109 - 0.193) | 1.426 (1.307 - 1.555) | 0.077 (0.011 - 0.539) | 383 |
| 60-69 | 9.1% | 1.000 (0.943 - 1) | 0.105 (0.079 - 0.139) | 1.000 (0.918 - 1) | 0.147 (0.117 - 0.184) | 1.118 (1.081 - 1.156) | - | 471 |
| 70-79 | 3.7% | 1.000 (0.942 - 1) | 0.045 (0.026 - 0.077) | 1.000 (0.758 - 1) | 0.196 (0.156 - 0.244) | 1.047 (1.02 - 1.075) | - | 328 |
| ≥80 | 2.5% | 1.000 (0.851 - 1) | 0.031 (0.011 - 0.088) | 1.000 (0.439 - 1) | 0.191 (0.13 - 0.273) | 1.032 (0.996 - 1.07) | - | 118 |
| Female patients | |  |  |  |  |  |  |  |
| <50 | 80.2% | 1.000 (0.207 - 1) | 0.812 (0.716 - 0.881) | 1.000 (0.947 - 1) | 0.059 (0.01 - 0.27) | 5.313 (3.416 - 8.261) | - | 86 |
| 50-59 | 58.0% | 1.000 (0.646 - 1) | 0.597 (0.534 - 0.656) | 1.000 (0.974 - 1) | 0.067 (0.033 - 0.131) | 2.480 (2.128 - 2.889) | - | 250 |
| 60-69 | 35.0% | 0.941 (0.73 - 0.99) | 0.367 (0.319 - 0.419) | 0.992 (0.958 - 0.999) | 0.067 (0.041 - 0.106) | 1.487 (1.289 - 1.716) | 0.160 (0.024 - 1.078) | 371 |
| 70-79 | 23.9% | 1.000 (0.851 - 1) | 0.257 (0.214 - 0.306) | 1.000 (0.958 - 1) | 0.080 (0.053 - 0.118) | 1.346 (1.265 - 1.433) | - | 364 |
| ≥80 | 9.3% | 1.000 (0.832 - 1) | 0.107 (0.064 - 0.175) | 1.000 (0.772 - 1) | 0.150 (0.098 - 0.222) | 1.120 (1.053 - 1.192) | - | 140 |

Table displays the test characteristics of ZCS for predicting ≥10% ischemia. Prevalence denotes the proportion of patients with ZCS in each group. Due to low number of cases, the first two age groups were taken together in women. NPV: negative predictive values. PPV: positive predictive value. PLR: positive likelihood ration. NLR: negative likely hood ratio. ZCS: zero calcium score.

Supplemental table S7: Test performance of different CACS cut-offs to detect abnormal PET (all patients, stratified by gender and diabetes)

| CACS cut-off | Sensitivity | Specificity | NPV | PPV |
| --- | --- | --- | --- | --- |
| **All patients (n = 2640)** | | | | |
| CACS ≥1 | 0.968 (0.95 - 0.979) | 0.322 (0.302 - 0.342) | 0.974 (0.959 - 0.983) | 0.277 (0.257 - 0.297) |
| CACS ≥5 | 0.955 (0.935 - 0.969) | 0.394 (0.373 - 0.415) | 0.970 (0.957 - 0.98) | 0.297 (0.276 - 0.318) |
| CACS ≥10 | 0.932 (0.908 - 0.95) | 0.436 (0.414 - 0.457) | 0.960 (0.945 - 0.971) | 0.307 (0.285 - 0.329) |
| CACS ≥20 | 0.907 (0.88 - 0.928) | 0.473 (0.452 - 0.495) | 0.950 (0.935 - 0.962) | 0.316 (0.293 - 0.339) |
| CACS ≥100 | 0.790 (0.755 - 0.822) | 0.653 (0.633 - 0.673) | 0.921 (0.906 - 0.933) | 0.379 (0.352 - 0.407) |
| **Male patients (n = 1429)** | | | | |
| CACS ≥1 | 0.978 (0.959 - 0.988) | 0.228 (0.203 - 0.255) | 0.963 (0.931 - 0.98) | 0.335 (0.309 - 0.363) |
| CACS ≥5 | 0.968 (0.946 - 0.981) | 0.297 (0.27 - 0.326) | 0.959 (0.931 - 0.976) | 0.354 (0.327 - 0.383) |
| CACS ≥10 | 0.939 (0.911 - 0.958) | 0.341 (0.313 - 0.371) | 0.933 (0.903 - 0.954) | 0.362 (0.334 - 0.392) |
| CACS ≥20 | 0.926 (0.897 - 0.948) | 0.381 (0.351 - 0.411) | 0.928 (0.9 - 0.949) | 0.373 (0.344 - 0.404) |
| CACS ≥100 | 0.806 (0.765 - 0.841) | 0.561 (0.53 - 0.591) | 0.879 (0.852 - 0.902) | 0.422 (0.388 - 0.457) |
| **Female patients (n = 1211)** | | | | |
| CACS ≥1 | 0.940 (0.891 - 0.968) | 0.412 (0.383 - 0.442) | 0.980 (0.962 - 0.989) | 0.186 (0.16 - 0.215) |
| CACS ≥5 | 0.921 (0.866 - 0.954) | 0.487 (0.457 - 0.517) | 0.977 (0.961 - 0.987) | 0.204 (0.175 - 0.235) |
| CACS ≥10 | 0.914 (0.858 - 0.949) | 0.526 (0.496 - 0.556) | 0.977 (0.961 - 0.987) | 0.216 (0.186 - 0.249) |
| CACS ≥20 | 0.854 (0.789 - 0.902) | 0.562 (0.532 - 0.592) | 0.964 (0.947 - 0.976) | 0.218 (0.186 - 0.253) |
| CACS ≥100 | 0.748 (0.674 - 0.811) | 0.742 (0.715 - 0.768) | 0.954 (0.937 - 0.966) | 0.293 (0.25 - 0.34) |
| **Diabetic patients (n = 635)** | | | | |
| CACS ≥1 | 0.982 (0.95 - 0.994) | 0.250 (0.213 - 0.291) | 0.975 (0.928 - 0.991) | 0.326 (0.287 - 0.367) |
| CACS ≥5 | 0.971 (0.933 - 0.987) | 0.317 (0.276 - 0.36) | 0.967 (0.925 - 0.986) | 0.344 (0.303 - 0.387) |
| CACS ≥10 | 0.947 (0.903 - 0.972) | 0.358 (0.315 - 0.402) | 0.949 (0.905 - 0.973) | 0.352 (0.31 - 0.397) |
| CACS ≥20 | 0.936 (0.888 - 0.964) | 0.405 (0.361 - 0.45) | 0.945 (0.904 - 0.969) | 0.367 (0.323 - 0.413) |
| CACS ≥100 | 0.877 (0.82 - 0.918) | 0.591 (0.545 - 0.634) | 0.929 (0.894 - 0.953) | 0.441 (0.389 - 0.494) |
| **Non-diabetic patients (n = 2005)** | | | | |
| CACS ≥1 | 0.961 (0.937 - 0.976) | 0.342 (0.32 - 0.366) | 0.974 (0.957 - 0.984) | 0.259 (0.237 - 0.282) |
| CACS ≥5 | 0.948 (0.922 - 0.966) | 0.416 (0.392 - 0.44) | 0.971 (0.956 - 0.981) | 0.280 (0.256 - 0.305) |
| CACS ≥10 | 0.925 (0.894 - 0.947) | 0.458 (0.434 - 0.482) | 0.962 (0.946 - 0.974) | 0.290 (0.265 - 0.316) |
| CACS ≥20 | 0.894 (0.859 - 0.921) | 0.493 (0.468 - 0.517) | 0.951 (0.934 - 0.964) | 0.296 (0.271 - 0.323) |
| CACS ≥100 | 0.752 (0.707 - 0.792) | 0.671 (0.648 - 0.694) | 0.919 (0.902 - 0.933) | 0.354 (0.322 - 0.387) |
| **Diabetic males (n = 372)** | | | | |
| CACS ≥1 | 0.977 (0.934 - 0.992) | 0.169 (0.127 - 0.221) | 0.932 (0.818 - 0.977) | 0.384 (0.333 - 0.438) |
| CACS ≥5 | 0.969 (0.923 - 0.988) | 0.239 (0.189 - 0.296) | 0.935 (0.846 - 0.975) | 0.403 (0.35 - 0.459) |
| CACS ≥10 | 0.938 (0.882 - 0.968) | 0.292 (0.239 - 0.352) | 0.899 (0.813 - 0.948) | 0.413 (0.358 - 0.47) |
| CACS ≥20 | 0.930 (0.873 - 0.963) | 0.342 (0.285 - 0.403) | 0.902 (0.824 - 0.948) | 0.429 (0.372 - 0.487) |
| CACS ≥100 | 0.876 (0.808 - 0.922) | 0.523 (0.46 - 0.585) | 0.888 (0.826 - 0.93) | 0.493 (0.429 - 0.558) |
| **Non-diabetic males (n = 1057)** | | | | |
| CACS ≥1 | 0.978 (0.954 - 0.99) | 0.246 (0.217 - 0.278) | 0.970 (0.935 - 0.986) | 0.317 (0.286 - 0.349) |
| CACS ≥5 | 0.968 (0.94 - 0.983) | 0.316 (0.284 - 0.349) | 0.965 (0.934 - 0.981) | 0.335 (0.304 - 0.369) |
| CACS ≥10 | 0.939 (0.904 - 0.961) | 0.357 (0.324 - 0.391) | 0.942 (0.91 - 0.964) | 0.343 (0.31 - 0.377) |
| CACS ≥20 | 0.924 (0.887 - 0.95) | 0.393 (0.359 - 0.428) | 0.936 (0.904 - 0.958) | 0.352 (0.318 - 0.387) |
| CACS ≥100 | 0.773 (0.721 - 0.819) | 0.573 (0.538 - 0.607) | 0.876 (0.845 - 0.902) | 0.392 (0.352 - 0.434) |
| **Diabetic females (n = 263)** | | | | |
| CACS ≥1 | 1.000 (0.916 - 1) | 0.403 (0.34 - 0.469) | 1.000 (0.959 - 1) | 0.241 (0.184 - 0.31) |
| CACS ≥5 | 0.976 (0.877 - 0.996) | 0.403 (0.34 - 0.469) | 0.989 (0.94 - 0.998) | 0.237 (0.18 - 0.306) |
| CACS ≥10 | 0.976 (0.877 - 0.996) | 0.430 (0.366 - 0.496) | 0.990 (0.943 - 0.998) | 0.246 (0.186 - 0.316) |
| CACS ≥20 | 0.952 (0.842 - 0.987) | 0.475 (0.41 - 0.541) | 0.981 (0.934 - 0.995) | 0.256 (0.194 - 0.33) |
| CACS ≥100 | 0.881 (0.75 - 0.948) | 0.665 (0.601 - 0.724) | 0.967 (0.925 - 0.986) | 0.333 (0.253 - 0.425) |
| **Non-diabetic females (n = 948)** | | | | |
| CACS ≥1 | 0.917 (0.85 - 0.956) | 0.431 (0.398 - 0.465) | 0.976 (0.955 - 0.987) | 0.173 (0.145 - 0.206) |
| CACS ≥5 | 0.899 (0.828 - 0.943) | 0.509 (0.475 - 0.543) | 0.975 (0.956 - 0.986) | 0.192 (0.16 - 0.229) |
| CACS ≥10 | 0.890 (0.817 - 0.936) | 0.552 (0.518 - 0.585) | 0.975 (0.956 - 0.985) | 0.205 (0.171 - 0.244) |
| CACS ≥20 | 0.817 (0.734 - 0.878) | 0.585 (0.552 - 0.618) | 0.961 (0.94 - 0.975) | 0.204 (0.169 - 0.244) |
| CACS ≥100 | 0.697 (0.605 - 0.776) | 0.763 (0.733 - 0.79) | 0.951 (0.932 - 0.965) | 0.276 (0.227 - 0.332) |

Table indicates test performance of different CACS cut-offs to detect abnormal PET (defined SSS ≥4). Number in brackets indicate 95%-confidence interval. NPV: negative predictive value. PPV: positive predictive value.

Supplemental table S8: Test performance of different CACS cut-offs to detect abnormal PET (all patients, stratified by age group)

| CACS cut-off | Sensitivity | Specificity | NPV | PPV |
| --- | --- | --- | --- | --- |
| **All patients <40 (n = 45)** | | | | |
| CACS ≥1 | 1.000 (0.207 - 1) | 0.091 (0.036 - 0.212) | 1.000 (0.51 - 1) | 0.024 (0.004 - 0.126) |
| CACS ≥5 | 1.000 (0.207 - 1) | 0.068 (0.023 - 0.182) | 1.000 (0.439 - 1) | 0.024 (0.004 - 0.123) |
| CACS ≥10 | 1.000 (0.207 - 1) | 0.068 (0.023 - 0.182) | 1.000 (0.439 - 1) | 0.024 (0.004 - 0.123) |
| CACS ≥20 | 1.000 (0.207 - 1) | 0.932 (0.818 - 0.977) | 1.000 (0.914 - 1) | 0.250 (0.046 - 0.699) |
| CACS ≥100 | 1.000 (0.207 - 1) | 0.977 (0.882 - 0.996) | 1.000 (0.918 - 1) | 0.500 (0.095 - 0.905) |
| **All patients 40-49 (n = 170)** | | | | |
| CACS ≥1 | 0.923 (0.667 - 0.986) | 0.656 (0.579 - 0.726) | 0.990 (0.948 - 0.998) | 0.182 (0.107 - 0.291) |
| CACS ≥5 | 0.846 (0.578 - 0.957) | 0.732 (0.658 - 0.796) | 0.983 (0.94 - 0.995) | 0.208 (0.12 - 0.335) |
| CACS ≥10 | 0.846 (0.578 - 0.957) | 0.758 (0.685 - 0.818) | 0.983 (0.942 - 0.995) | 0.224 (0.13 - 0.359) |
| CACS ≥20 | 0.692 (0.424 - 0.873) | 0.796 (0.726 - 0.852) | 0.969 (0.923 - 0.988) | 0.220 (0.12 - 0.367) |
| CACS ≥100 | 0.385 (0.177 - 0.645) | 0.898 (0.841 - 0.936) | 0.946 (0.898 - 0.973) | 0.238 (0.106 - 0.451) |
| **All patients 50-59 (n = 633)** | | | | |
| CACS ≥1 | 0.934 (0.864 - 0.969) | 0.458 (0.416 - 0.5) | 0.976 (0.949 - 0.989) | 0.224 (0.185 - 0.269) |
| CACS ≥5 | 0.923 (0.85 - 0.962) | 0.563 (0.521 - 0.604) | 0.978 (0.954 - 0.989) | 0.262 (0.217 - 0.312) |
| CACS ≥10 | 0.879 (0.796 - 0.931) | 0.624 (0.582 - 0.663) | 0.968 (0.944 - 0.982) | 0.282 (0.233 - 0.337) |
| CACS ≥20 | 0.846 (0.758 - 0.906) | 0.649 (0.608 - 0.688) | 0.962 (0.937 - 0.977) | 0.288 (0.237 - 0.345) |
| CACS ≥100 | 0.637 (0.535 - 0.729) | 0.817 (0.783 - 0.848) | 0.931 (0.904 - 0.95) | 0.369 (0.298 - 0.447) |
| **All patients 60-69 (n = 842)** | | | | |
| CACS ≥1 | 0.972 (0.937 - 0.988) | 0.256 (0.224 - 0.29) | 0.971 (0.935 - 0.988) | 0.263 (0.231 - 0.298) |
| CACS ≥5 | 0.945 (0.901 - 0.97) | 0.321 (0.286 - 0.357) | 0.955 (0.919 - 0.975) | 0.276 (0.242 - 0.312) |
| CACS ≥10 | 0.923 (0.874 - 0.953) | 0.360 (0.324 - 0.397) | 0.944 (0.909 - 0.967) | 0.283 (0.248 - 0.321) |
| CACS ≥20 | 0.901 (0.848 - 0.936) | 0.415 (0.378 - 0.452) | 0.938 (0.905 - 0.961) | 0.296 (0.26 - 0.336) |
| CACS ≥100 | 0.785 (0.719 - 0.838) | 0.610 (0.572 - 0.646) | 0.912 (0.882 - 0.935) | 0.355 (0.31 - 0.403) |
| **All patients 70-79 (n = 692)** | | | | |
| CACS ≥1 | 0.966 (0.927 - 0.984) | 0.182 (0.151 - 0.217) | 0.940 (0.875 - 0.972) | 0.285 (0.251 - 0.323) |
| CACS ≥5 | 0.966 (0.927 - 0.984) | 0.238 (0.203 - 0.276) | 0.953 (0.902 - 0.979) | 0.300 (0.264 - 0.339) |
| CACS ≥10 | 0.943 (0.898 - 0.969) | 0.271 (0.234 - 0.311) | 0.933 (0.882 - 0.963) | 0.304 (0.267 - 0.344) |
| CACS >20 | 0.926 (0.877 - 0.956) | 0.306 (0.267 - 0.347) | 0.924 (0.874 - 0.955) | 0.311 (0.273 - 0.352) |
| CACS ≥100 | 0.846 (0.785 - 0.892) | 0.507 (0.464 - 0.55) | 0.907 (0.867 - 0.935) | 0.367 (0.322 - 0.415) |
| **All patients ≥80 (n = 258)** | | | | |
| CACS ≥1 | 1.000 (0.962 - 1) | 0.099 (0.062 - 0.155) | 1.000 (0.806 - 1) | 0.401 (0.341 - 0.464) |
| CACS ≥5 | 0.990 (0.944 - 0.998) | 0.149 (0.102 - 0.212) | 0.960 (0.805 - 0.993) | 0.412 (0.351 - 0.476) |
| CACS ≥10 | 0.990 (0.944 - 0.998) | 0.193 (0.139 - 0.26) | 0.969 (0.843 - 0.994) | 0.425 (0.362 - 0.49) |
| CACS >20 | 0.969 (0.913 - 0.989) | 0.217 (0.161 - 0.287) | 0.921 (0.792 - 0.973) | 0.427 (0.364 - 0.493) |
| CACS ≥100 | 0.897 (0.821 - 0.943) | 0.422 (0.349 - 0.5) | 0.872 (0.78 - 0.929) | 0.483 (0.411 - 0.556) |

Table indicates test performance of different CACS cut-offs to detect abnormal PET (defined SSS ≥4). Number in brackets indicate 95%-confidence interval. NPV: negative predictive value. PPV: positive predictive value.

Supplemental table S9: Test performance of different CACS cut-offs to detect abnormal PET (male patients, stratified by age group)

| CACS cut-off | Sensitivity | Specificity | NPV | PPV |
| --- | --- | --- | --- | --- |
| **Male patients <40 (n = 26)** | | | | |
| CACS ≥1 | 1.000 (0.207 - 1) | 0.840 (0.653 - 0.936) | 1.000 (0.845 - 1) | 0.200 (0.036 - 0.624) |
| CACS ≥5 | 1.000 (0.207 - 1) | 0.880 (0.7 - 0.958) | 1.000 (0.851 - 1) | 0.250 (0.046 - 0.699) |
| CACS ≥10 | 1.000 (0.207 - 1) | 0.880 (0.7 - 0.958) | 1.000 (0.851 - 1) | 0.250 (0.046 - 0.699) |
| CACS ≥20 | 1.000 (0.207 - 1) | 0.880 (0.7 - 0.958) | 1.000 (0.851 - 1) | 0.250 (0.046 - 0.699) |
| CACS ≥100 | 1.000 (0.207 - 1) | 0.960 (0.805 - 0.993) | 1.000 (0.862 - 1) | 0.500 (0.095 - 0.905) |
| **Male patients 40-49 (n = 103)** | | | | |
| CACS ≥1 | 0.909 (0.623 - 0.984) | 0.576 (0.474 - 0.672) | 0.981 (0.902 - 0.997) | 0.204 (0.115 - 0.336) |
| CACS ≥5 | 0.909 (0.623 - 0.984) | 0.663 (0.562 - 0.751) | 0.984 (0.914 - 0.997) | 0.244 (0.138 - 0.393) |
| CACS ≥10 | 0.818 (0.523 - 0.949) | 0.685 (0.584 - 0.771) | 0.969 (0.895 - 0.992) | 0.237 (0.13 - 0.392) |
| CACS ≥20 | 0.727 (0.434 - 0.903) | 0.739 (0.641 - 0.818) | 0.958 (0.883 - 0.986) | 0.250 (0.133 - 0.421) |
| CACS ≥100 | 0.364 (0.152 - 0.646) | 0.848 (0.761 - 0.907) | 0.918 (0.84 - 0.96) | 0.222 (0.09 - 0.452) |
| **Male patients 50-59 (n= 383)** | | | | |
| CACS ≥1 | 0.935 (0.857 - 0.972) | 0.340 (0.289 - 0.395) | 0.954 (0.897 - 0.98) | 0.263 (0.214 - 0.318) |
| CACS ≥5 | 0.922 (0.84 - 0.964) | 0.448 (0.393 - 0.504) | 0.958 (0.911 - 0.981) | 0.296 (0.242 - 0.356) |
| CACS ≥10 | 0.870 (0.777 - 0.928) | 0.520 (0.464 - 0.575) | 0.941 (0.895 - 0.968) | 0.313 (0.255 - 0.378) |
| CACS ≥20 | 0.844 (0.747 - 0.909) | 0.552 (0.496 - 0.607) | 0.934 (0.888 - 0.962) | 0.322 (0.261 - 0.389) |
| CACS ≥100 | 0.623 (0.512 - 0.723) | 0.765 (0.714 - 0.809) | 0.890 (0.846 - 0.922) | 0.400 (0.317 - 0.489) |
| **Male patients 60-69 (n = 471)** | | | | |
| CACS ≥1 | 0.993 (0.961 - 0.999) | 0.127 (0.095 - 0.167) | 0.977 (0.879 - 0.996) | 0.325 (0.282 - 0.371) |
| CACS ≥5 | 0.979 (0.939 - 0.993) | 0.172 (0.135 - 0.217) | 0.950 (0.863 - 0.983) | 0.333 (0.289 - 0.38) |
| CACS ≥10 | 0.957 (0.91 - 0.98) | 0.215 (0.174 - 0.262) | 0.922 (0.84 - 0.964) | 0.340 (0.295 - 0.388) |
| CACS ≥20 | 0.943 (0.891 - 0.971) | 0.269 (0.224 - 0.319) | 0.918 (0.846 - 0.958) | 0.353 (0.306 - 0.403) |
| CACS ≥100 | 0.836 (0.766 - 0.888) | 0.462 (0.409 - 0.516) | 0.869 (0.812 - 0.911) | 0.397 (0.342 - 0.453) |
| **Male patients 70-79 (n =328)** | | | | |
| CACS ≥1 | 0.983 (0.942 - 0.995) | 0.048 (0.026 - 0.087) | 0.833 (0.552 - 0.953) | 0.377 (0.325 - 0.431) |
| CACS ≥5 | 0.983 (0.942 - 0.995) | 0.101 (0.067 - 0.15) | 0.913 (0.732 - 0.976) | 0.390 (0.337 - 0.446) |
| CACS ≥10 | 0.950 (0.896 - 0.977) | 0.135 (0.095 - 0.189) | 0.824 (0.665 - 0.917) | 0.391 (0.337 - 0.448) |
| CACS ≥20 | 0.950 (0.896 - 0.977) | 0.169 (0.124 - 0.226) | 0.854 (0.716 - 0.931) | 0.401 (0.346 - 0.458) |
| CACS ≥100 | 0.884 (0.815 - 0.93) | 0.333 (0.273 - 0.4) | 0.831 (0.737 - 0.897) | 0.437 (0.376 - 0.499) |
| **Male patients ≥80 (n = 118)** | | | | |
| CACS ≥1 | 1.000 (0.937 - 1) | 0.049 (0.017 - 0.135) | 1.000 (0.439 - 1) | 0.496 (0.406 - 0.586) |
| CACS ≥5 | 0.982 (0.907 - 0.997) | 0.098 (0.046 - 0.198) | 0.857 (0.487 - 0.974) | 0.505 (0.413 - 0.596) |
| CACS ≥10 | 0.982 (0.907 - 0.997) | 0.098 (0.046 - 0.198) | 0.857 (0.487 - 0.974) | 0.505 (0.413 - 0.596) |
| CACS ≥20 | 0.982 (0.907 - 0.997) | 0.098 (0.046 - 0.198) | 0.857 (0.487 - 0.974) | 0.505 (0.413 - 0.596) |
| CACS ≥100 | 0.895 (0.789 - 0.951) | 0.246 (0.155 - 0.367) | 0.714 (0.5 - 0.862) | 0.526 (0.427 - 0.622) |

Table indicates test performance of different CACS cut-offs to detect abnormal PET (defined SSS ≥4). Number in brackets indicate 95%-confidence interval. NPV: negative predictive value. PPV: positive predictive value.

Supplemental table S10: Test performance of different CACS cut-offs to detect abnormal PET (female patients, stratified by age group)

| CACS cut-off | Sensitivity | Specificity | NPV | PPV |
| --- | --- | --- | --- | --- |
| **Female patients <50 (n = 86)** | | | | |
| CACS ≥1 | 1.000 (0.342 - 1) | 0.821 (0.726 - 0.889) | 1.000 (0.947 - 1) | 0.118 (0.033 - 0.343) |
| CACS ≥5 | 1.000 (0.342 - 1) | 0.869 (0.781 - 0.925) | 1.000 (0.95 - 1) | 0.154 (0.043 - 0.422) |
| CACS ≥10 | 1.000 (0.342 - 1) | 0.893 (0.809 - 0.943) | 1.000 (0.951 - 1) | 0.182 (0.051 - 0.477) |
| CACS ≥20 | 0.500 (0.095 - 0.905) | 0.905 (0.823 - 0.951) | 0.987 (0.93 - 0.998) | 0.111 (0.02 - 0.435) |
| CACS ≥100 | 0.500 (0.095 - 0.905) | 0.976 (0.917 - 0.993) | 0.988 (0.935 - 0.998) | 0.333 (0.061 - 0.792) |
| **Female patients 50-59 (n = 250)** | | | | |
| CACS ≥1 | 0.929 (0.685 - 0.987) | 0.610 (0.547 - 0.67) | 0.993 (0.962 - 0.999) | 0.124 (0.074 - 0.2) |
| CACS ≥5 | 0.929 (0.685 - 0.987) | 0.712 (0.651 - 0.766) | 0.994 (0.967 - 0.999) | 0.160 (0.096 - 0.255) |
| CACS ≥10 | 0.929 (0.685 - 0.987) | 0.758 (0.7 - 0.809) | 0.994 (0.969 - 0.999) | 0.186 (0.112 - 0.292) |
| CACS ≥20 | 0.857 (0.601 - 0.96) | 0.775 (0.718 - 0.824) | 0.989 (0.961 - 0.997) | 0.185 (0.109 - 0.296) |
| CACS ≥100 | 0.714 (0.454 - 0.883) | 0.886 (0.839 - 0.92) | 0.981 (0.953 - 0.993) | 0.270 (0.154 - 0.43) |
| **Female patients 60-69 (n = 371)** | | | | |
| CACS ≥1 | 0.902 (0.775 - 0.961) | 0.385 (0.334 - 0.438) | 0.969 (0.924 - 0.988) | 0.154 (0.114 - 0.205) |
| CACS ≥5 | 0.829 (0.687 - 0.915) | 0.470 (0.417 - 0.524) | 0.957 (0.914 - 0.979) | 0.163 (0.119 - 0.219) |
| CACS ≥10 | 0.805 (0.66 - 0.898) | 0.506 (0.452 - 0.56) | 0.954 (0.912 - 0.977) | 0.168 (0.122 - 0.227) |
| CACS ≥20 | 0.756 (0.607 - 0.862) | 0.561 (0.507 - 0.613) | 0.949 (0.908 - 0.972) | 0.176 (0.127 - 0.239) |
| CACS ≥100 | 0.610 (0.457 - 0.743) | 0.758 (0.709 - 0.801) | 0.940 (0.905 - 0.963) | 0.238 (0.167 - 0.328) |
| **Female patients 70-79 (n = 364)** | | | | |
| CACS ≥1 | 0.926 (0.824 - 0.971) | 0.271 (0.225 - 0.323) | 0.955 (0.889 - 0.982) | 0.181 (0.14 - 0.231) |
| CACS ≥5 | 0.926 (0.824 - 0.971) | 0.329 (0.279 - 0.383) | 0.962 (0.907 - 0.985) | 0.194 (0.15 - 0.246) |
| CACS ≥10 | 0.926 (0.824 - 0.971) | 0.361 (0.31 - 0.416) | 0.966 (0.915 - 0.987) | 0.202 (0.156 - 0.256) |
| CACS ≥20 | 0.870 (0.756 - 0.936) | 0.397 (0.344 - 0.452) | 0.946 (0.893 - 0.974) | 0.201 (0.155 - 0.257) |
| CACS ≥100 | 0.759 (0.631 - 0.854) | 0.623 (0.567 - 0.675) | 0.937 (0.895 - 0.963) | 0.259 (0.197 - 0.333) |
| **Female patients ≥80 (n = 140)** | | | | |
| CACS ≥1 | 1.000 (0.912 - 1) | 0.130 (0.078 - 0.21) | 1.000 (0.772 - 1) | 0.315 (0.241 - 0.4) |
| CACS ≥5 | 1.000 (0.912 - 1) | 0.180 (0.117 - 0.267) | 1.000 (0.824 - 1) | 0.328 (0.251 - 0.415) |
| CACS ≥10 | 1.000 (0.912 - 1) | 0.250 (0.175 - 0.343) | 1.000 (0.867 - 1) | 0.348 (0.267 - 0.439) |
| CACS ≥20 | 0.950 (0.835 - 0.986) | 0.290 (0.21 - 0.385) | 0.935 (0.793 - 0.982) | 0.349 (0.266 - 0.442) |
| CACS ≥100 | 0.900 (0.769 - 0.96) | 0.530 (0.433 - 0.625) | 0.930 (0.833 - 0.972) | 0.434 (0.332 - 0.541) |

Table indicates test performance of different CACS cut-offs to detect abnormal PET (defined SSS ≥4). Number in brackets indicate 95%-confidence interval. NPV: negative predictive value. PPV: positive predictive value.

Supplemental Table S11: Difference in baseline characteristics of ZCS patients with normal and abnormal PET

|  | SSS<4 (n = 667) | SSS≥4 (n = 18) | p-value |
| --- | --- | --- | --- |
| male gender | 50% | 50% | 0.213 |
| age [years] | 57.8±11.5 | 64.2±10.5 | 0.020 |
| BMI [kg/m2] | 29.3±6.7 | 28.6±5.6 | 0.701 |
| diabetes | 17.4% | 16.7% | 1.000 |
| cholesterol | 40.9% | 44.4% | 0.811 |
| hypertension | 54.9% | 61.1% | 0.640 |
| family history | 26.8% | 22.2% | 0.792 |
| smoker | 43.3% | 55.6% | 0.341 |
| angina | 44.5% | 27.8% | 0.229 |
| dyspnoea | 60.6% | 55.6% | 0.808 |

Intergroup difference was calculated using t-test or Fisher’s exact test.

Supplemental Table S12: Patients with abnormal PET despite ZCS

| Symptoms | Risk factors | PET result | Clinical course |
| --- | --- | --- | --- |
| male (50y), atypical AP | severe dyslipidaemia (retrospectively) | no scar, antero-septo-apical ischemia (SDS 6) | severe stenosis of mid LAD, PCI with 1xDES |
| male (48y), atypical chest pain, significant risk factors | NIDDM, dyslipidaemia, AHT, former smoker | not transmural scar basal inferior (SRS 6) with large ischemia (SDS 9) | 2 vessel CAD with CTO of mid RCA and severe stenosis M2. Elective PCI to both lesions |
| female (68y), typical AP CCS II, SOB NYHA II, 2 weeks ago arm pain during several hours | former smoker | not transmural scar antero-apical (SRS 3) with ischemia (SDS 7) | no follow-up data, should have undergone angiogram |
| male (56y), SOB NYHA II, LVEF 38% (TTE) | IDDM, dyslipidaemia, former smoker | large ischemia (SDS 20), predominantly in LAD | non-obstructive CAD on angiogram |
| male (78y), SOB NYHA III, DDD-PM due to 3rd degree AV block | AHT, dyslipidaemia | small inferoapical scar (SRS 3) with minimal ischemia (SDS 2), most likely PM induced | conservative, no coronary events |
| female (55y), atypical CAD with spasm and long tortuous stenosis of LAD, history of NSTEMI | dyslipidaemia, family history, former smoker | no scar, focal anteroapical ischemia (SDS 4) | conservative approach with OMT |
| male (66y), atypical chest pain, SOB NYHA II, possible hypokinesia inferolateral | AHT, family history | not transmural anterobasal scar (SSS 4), no ischemia | conservative, no coronary events |
| female (73y), atypical chest pain, rheumatoid arthritis | dyslipidaemia | small anterobasal scar (SRS 2) with ischemia (SDS 3), good MFR, judged to be not due to CAD | conservative, no coronary events |
| male (57y), hypertensive cardiomyopathy, inferolateral hypokinesia | AHT, dyslipidaemia, former smoker | not transmural scar inferolateral (SSS 4), no ischemia, most likely due to fibrosis, e.g. myocarditis | conservative, no coronary events |
| male (66y), atypical chest pain, prior AVR (severe regurgitation), ICD-CRT | AHT, dyslipidaemia | no scar, inferolateral ischemia (SDS 4) | no angiogram, conservative, no coronary event |
| male (79y), repaired pentalogy of Fallot, cor pulmonale, DDDR-ICD | AHT, former smoker | apical and anterolateral scar (SSS 5), no ischemia | conservative, no coronary events |
| female (72y), atypical AP CCS II, permanent AF, prior stroke, possible lung cancer | AHT, dyslipidaemia | no scar, possible ischemia lateral (SDS 4), possibly artefactual (AF, RBBB) | conservative, no intervention, follow-up PET SDS 2 |
| female (72y), DCM, prior stroke, hypokinesia inferoapical | AHT, family history, former smoker | inferoapical scar (SSS 6) without ischemia (SDS 0) | conservative, most likely due to DCM or prior myocarditis |
| male (50y), atypical chest pain, SOB, multiple TIA, hypertensive heart disease | AHT, family history, former smoker | not transmural scar apical and inferolateral (SSS 9), no ischemia (SDS 1) | no follow-up data |
| male (53y), inferior/inferolateral akinesia on TTE, prior stroke | AHT, former smoker | not transmural scar inferolateral (SSS 6) without ischemia, low MBF suggesting MVD | CTCA normal, most likely embolic scar |
| female (69y), atypical chest pain, paroxysmal AF | AHT, dyslipidaemia | no scar, anterolateral ischemia, normal MFR, no CTCA possible | angiogram showed normal coronaries |
| male (79y), SOB NYHA II, ischemia on SPECT | NIDDM, AHT, former smoker | no scar, inferolateral ischemia | conservative, no coronary events |
| female (64y), typical AP CCS II, SOB NYHA II, HFpEF, anteroseptal hypokinesia on TTE | none | no scar, inferolateral ischemia (SDS 4), normal flow reserve | conservative, no coronary events |
|  |  |  |  |

Patients’ medical records were screened for sudden cardiac death or acute coronary syndrome. If there was no such event, patients were considered to have “no coronary event”. Prevalence of risk factors was the following: diabetes 17%, hypertension 61%, family history 22%, smoker 56% and elevated cholesterol in 44%.

Supplemental Figure S1: ROC analysis of calcium score for predicting abnormal PET (SSS ≥4)


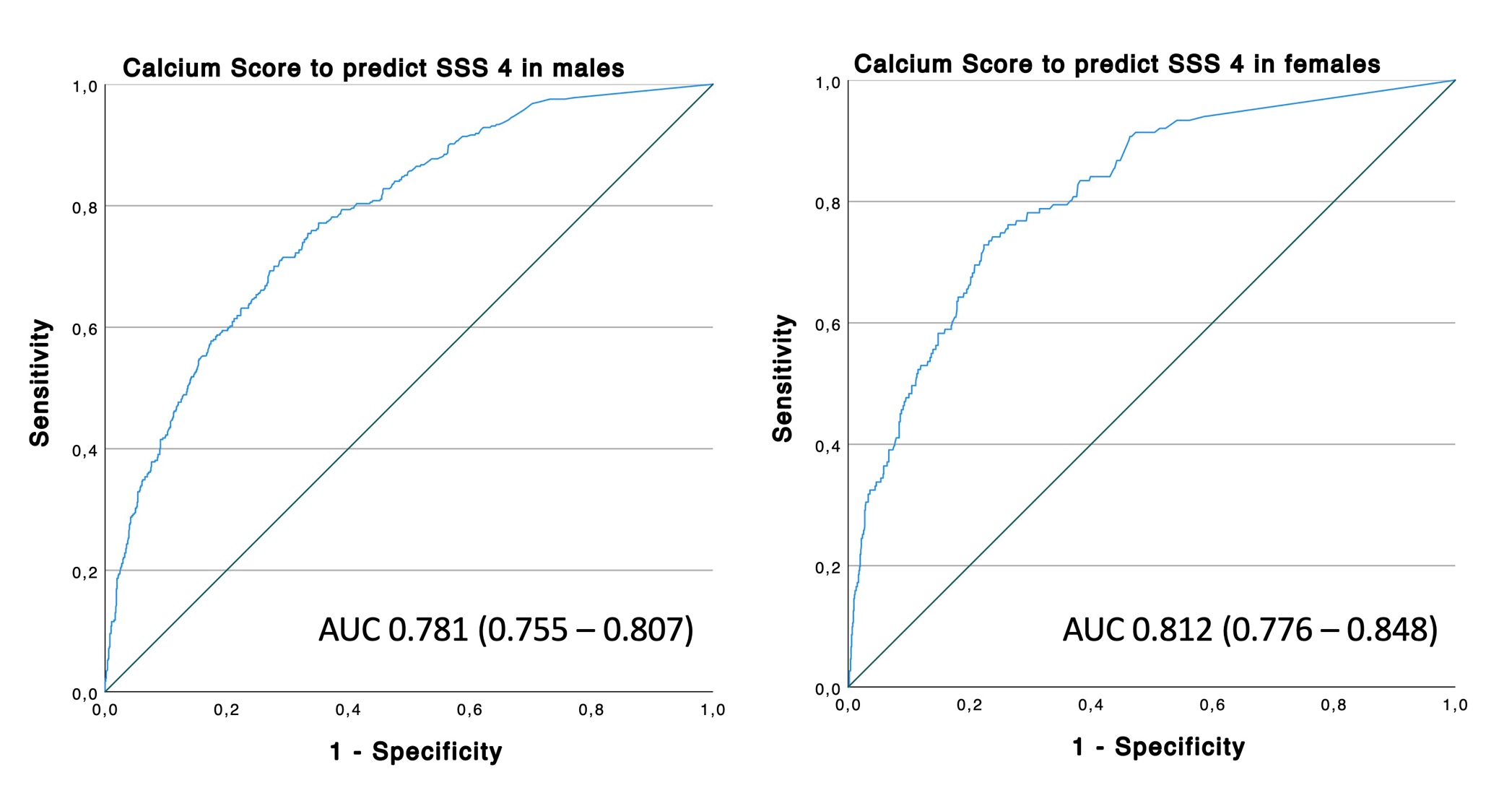


Supplemental Figure S2: ROC analysis of calcium score for predicting ≥10% ischemia (SDS ≥7)


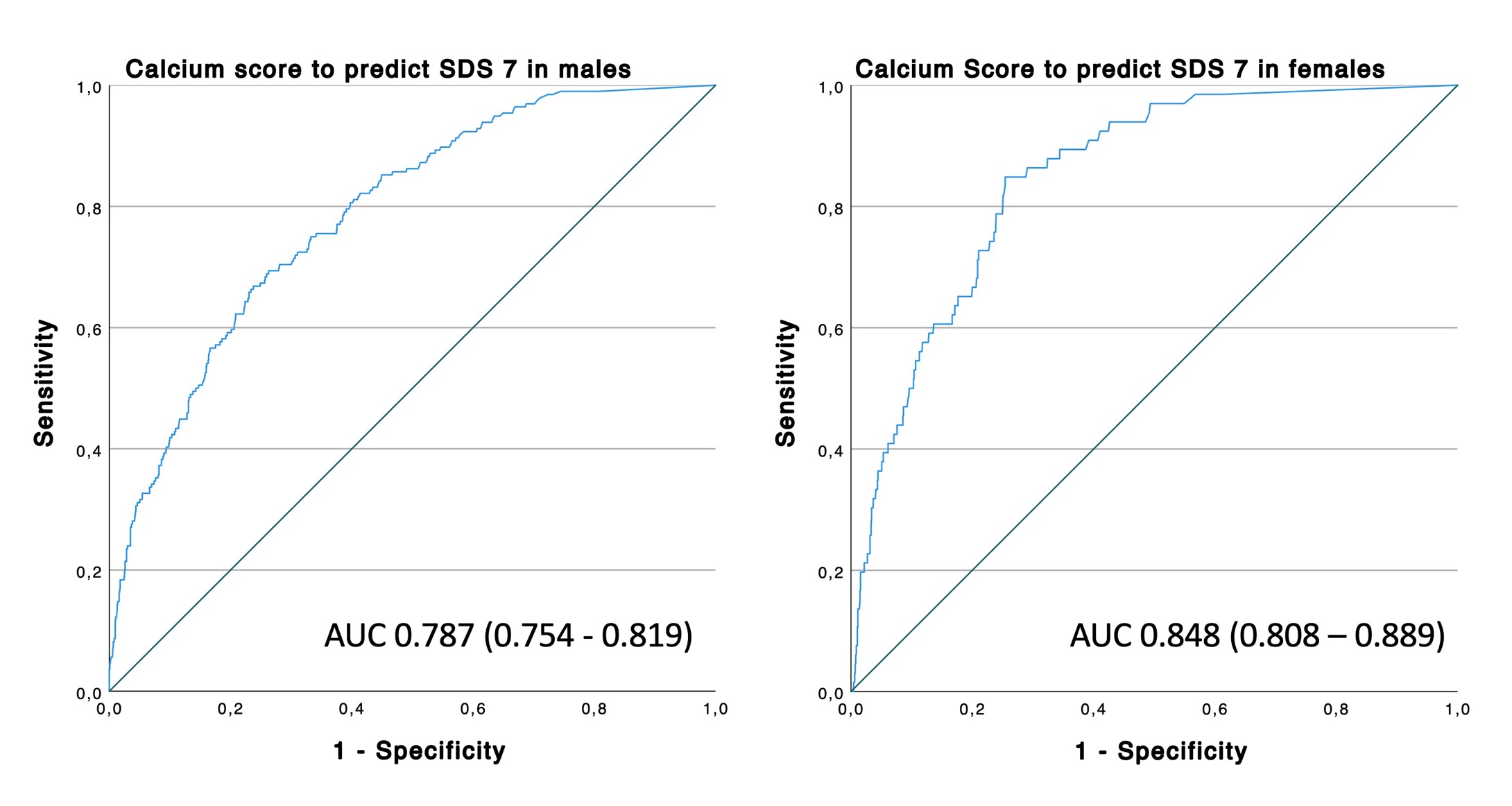

Supplement: Supplementary file 1 — Supplementary file1 (DOCX 528 kb) [file 12350_2022_3174_MOESM1_ESM.docx]
