# Supplementary material for: The power of zero calcium in 82-Rubidium PET irrespective of sex and age
Source: J Nucl Cardiol. 2023 Jan 9;30(4):1514–27. doi: 10.1007/s12350-022-03174-3 (PMC10371904; doi:10.1007/s12350-022-03174-3)
Supplement: Supplementary file 2 — Supplementary file2 (PPTX 1416 kb) [file 12350_2022_3174_MOESM2_ESM.pptx]

## Slide 1
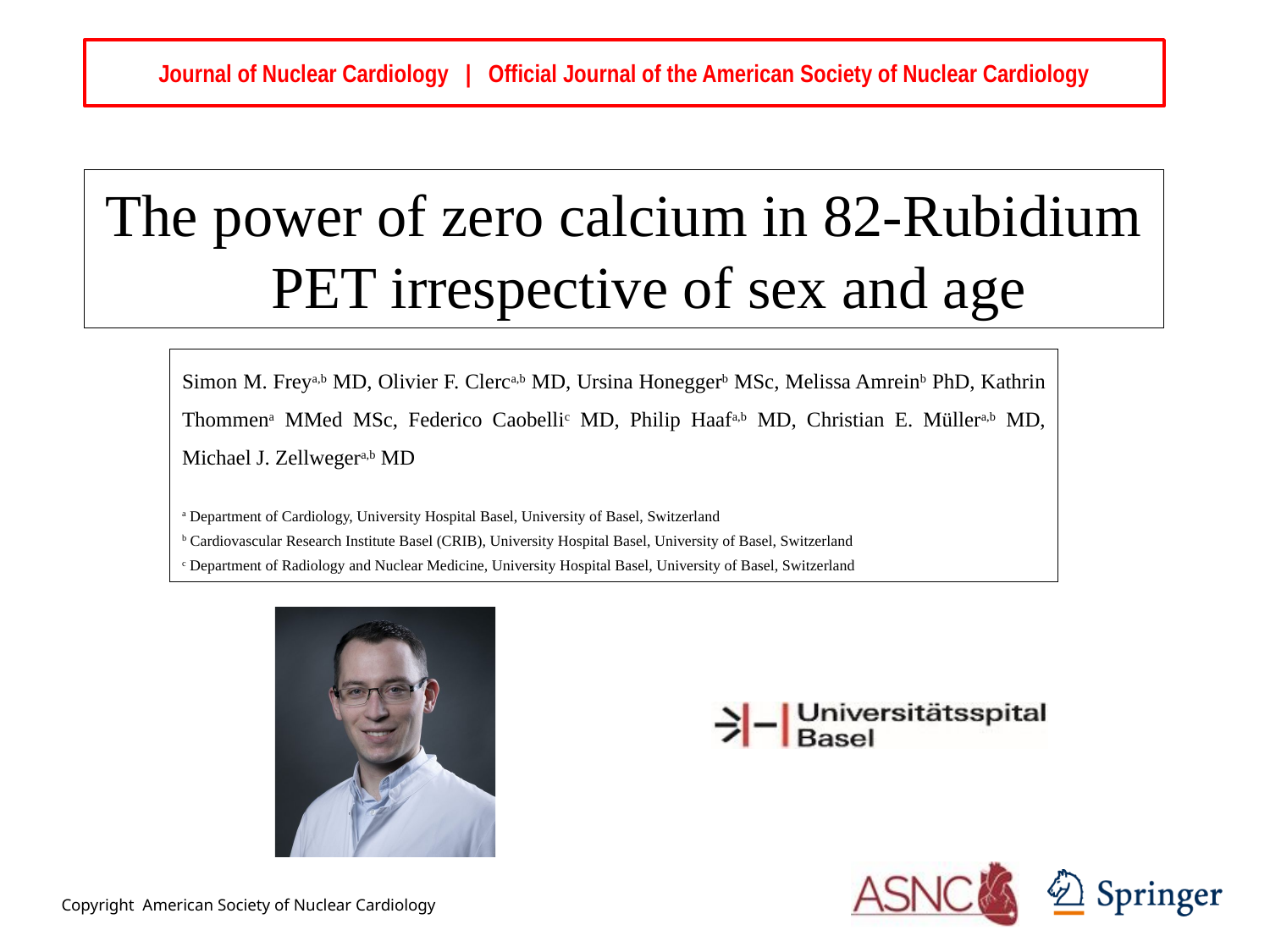

Journal of Nuclear Cardiology | Official Journal of the American Society of Nuclear Cardiology
# The power of zero calcium in 82-Rubidium PET irrespective of sex and age
Simon M. Freya,b MD, Olivier F. Clerca,b MD, Ursina Honeggerb MSc, Melissa Amreinb PhD, Kathrin Thommena MMed MSc, Federico Caobellic MD, Philip Haafa,b MD, Christian E. Müllera,b MD, Michael J. Zellwegera,b MD
a Department of Cardiology, University Hospital Basel, University of Basel, Switzerland
b Cardiovascular Research Institute Basel (CRIB), University Hospital Basel, University of Basel, Switzerland
c Department of Radiology and Nuclear Medicine, University Hospital Basel, University of Basel, Switzerland
Copyright American Society of Nuclear Cardiology

## Slide 2
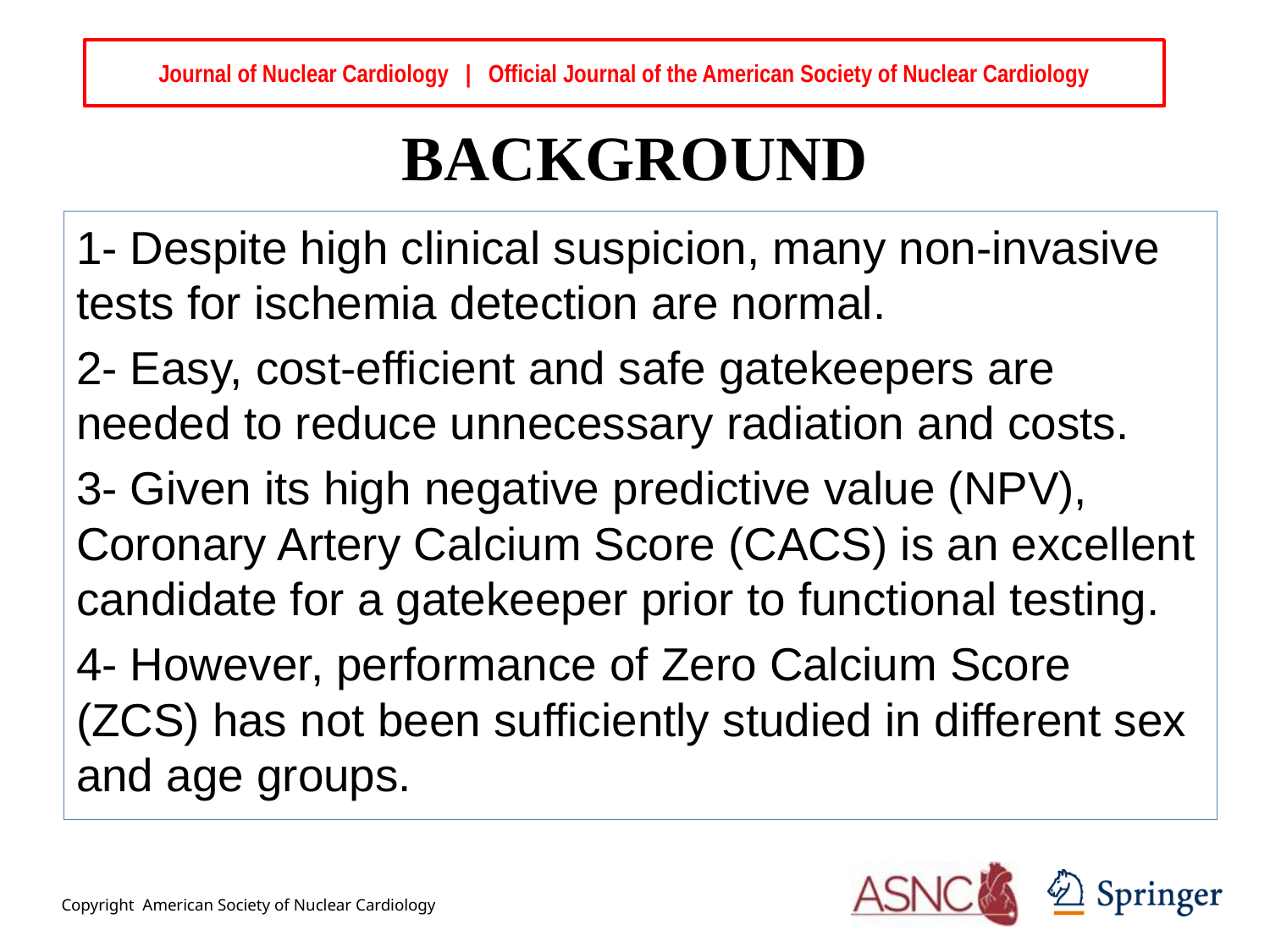

Journal of Nuclear Cardiology | Official Journal of the American Society of Nuclear Cardiology
# BACKGROUND
1- Despite high clinical suspicion, many non-invasive tests for ischemia detection are normal.
2- Easy, cost-efficient and safe gatekeepers are needed to reduce unnecessary radiation and costs.
3- Given its high negative predictive value (NPV), Coronary Artery Calcium Score (CACS) is an excellent candidate for a gatekeeper prior to functional testing.
4- However, performance of Zero Calcium Score (ZCS) has not been sufficiently studied in different sex and age groups.
Copyright American Society of Nuclear Cardiology

## Slide 3
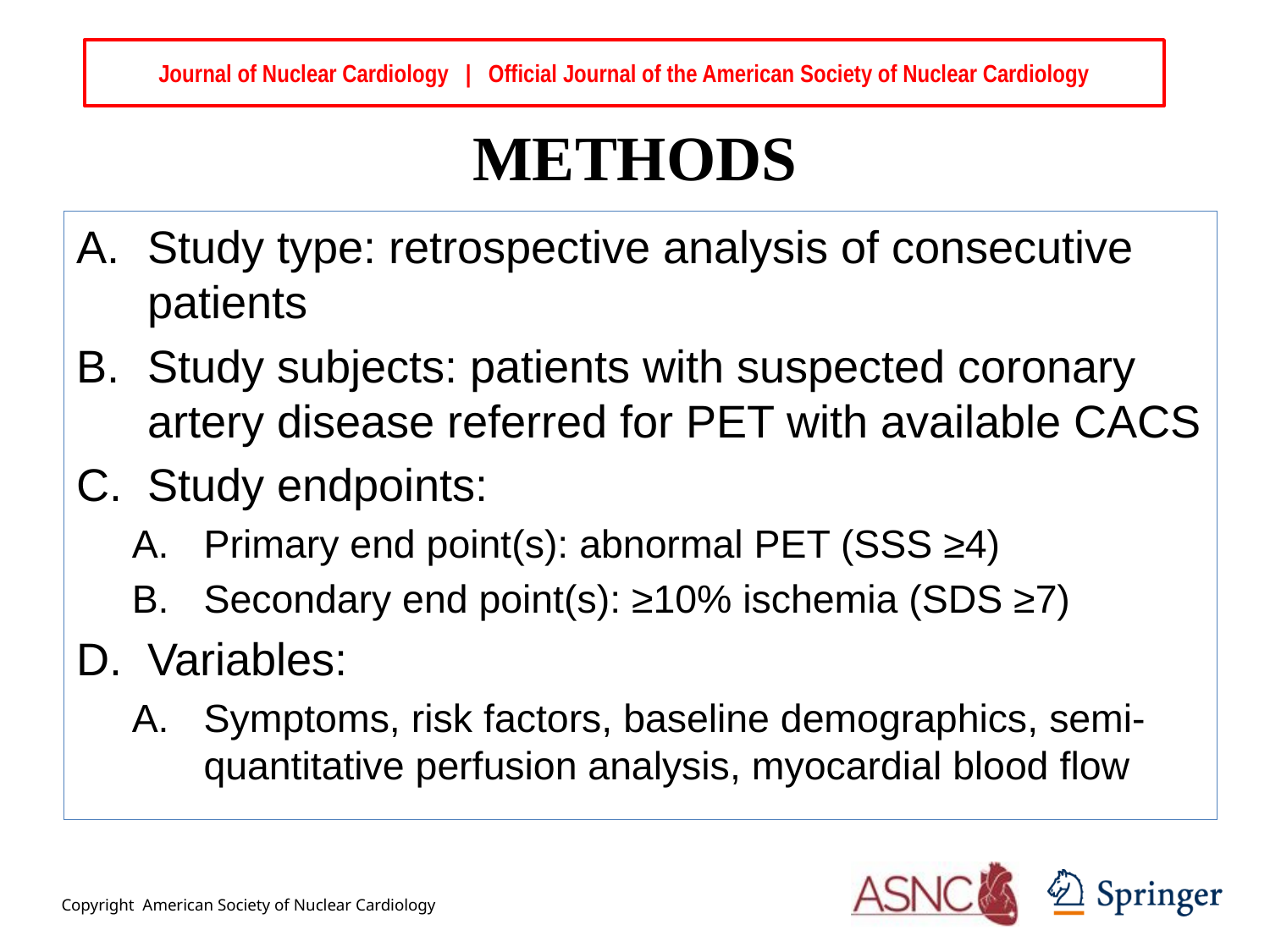

Journal of Nuclear Cardiology | Official Journal of the American Society of Nuclear Cardiology
# METHODS
Study type: retrospective analysis of consecutive patients
Study subjects: patients with suspected coronary artery disease referred for PET with available CACS
Study endpoints:
Primary end point(s): abnormal PET (SSS ≥4)
Secondary end point(s): ≥10% ischemia (SDS ≥7)
Variables:
Symptoms, risk factors, baseline demographics, semi-quantitative perfusion analysis, myocardial blood flow
Copyright American Society of Nuclear Cardiology

## Slide 4
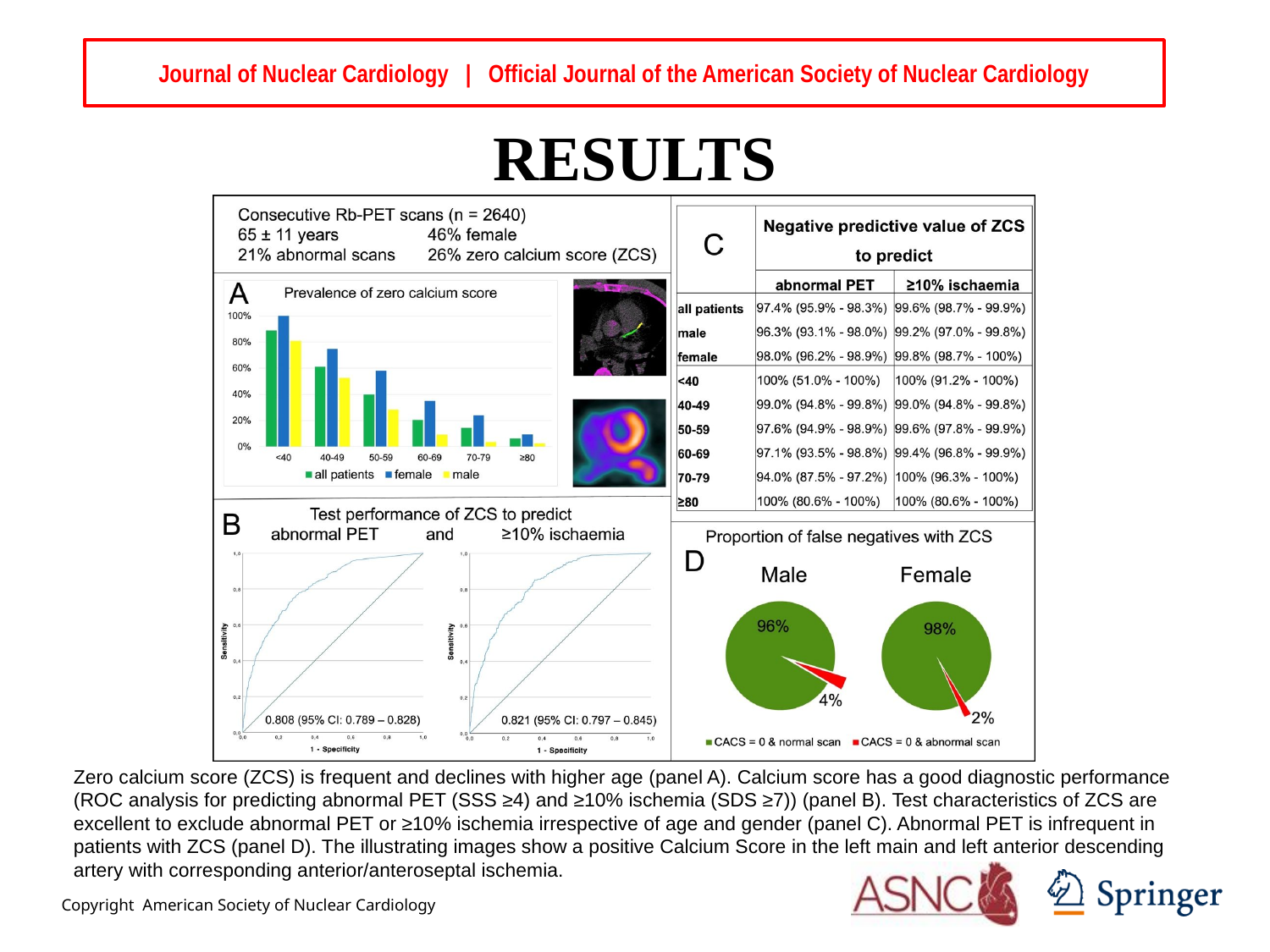

Journal of Nuclear Cardiology | Official Journal of the American Society of Nuclear Cardiology
# RESULTS
Zero calcium score (ZCS) is frequent and declines with higher age (panel A). Calcium score has a good diagnostic performance (ROC analysis for predicting abnormal PET (SSS ≥4) and ≥10% ischemia (SDS ≥7)) (panel B). Test characteristics of ZCS are excellent to exclude abnormal PET or ≥10% ischemia irrespective of age and gender (panel C). Abnormal PET is infrequent in patients with ZCS (panel D). The illustrating images show a positive Calcium Score in the left main and left anterior descending artery with corresponding anterior/anteroseptal ischemia.
Copyright American Society of Nuclear Cardiology

## Slide 5
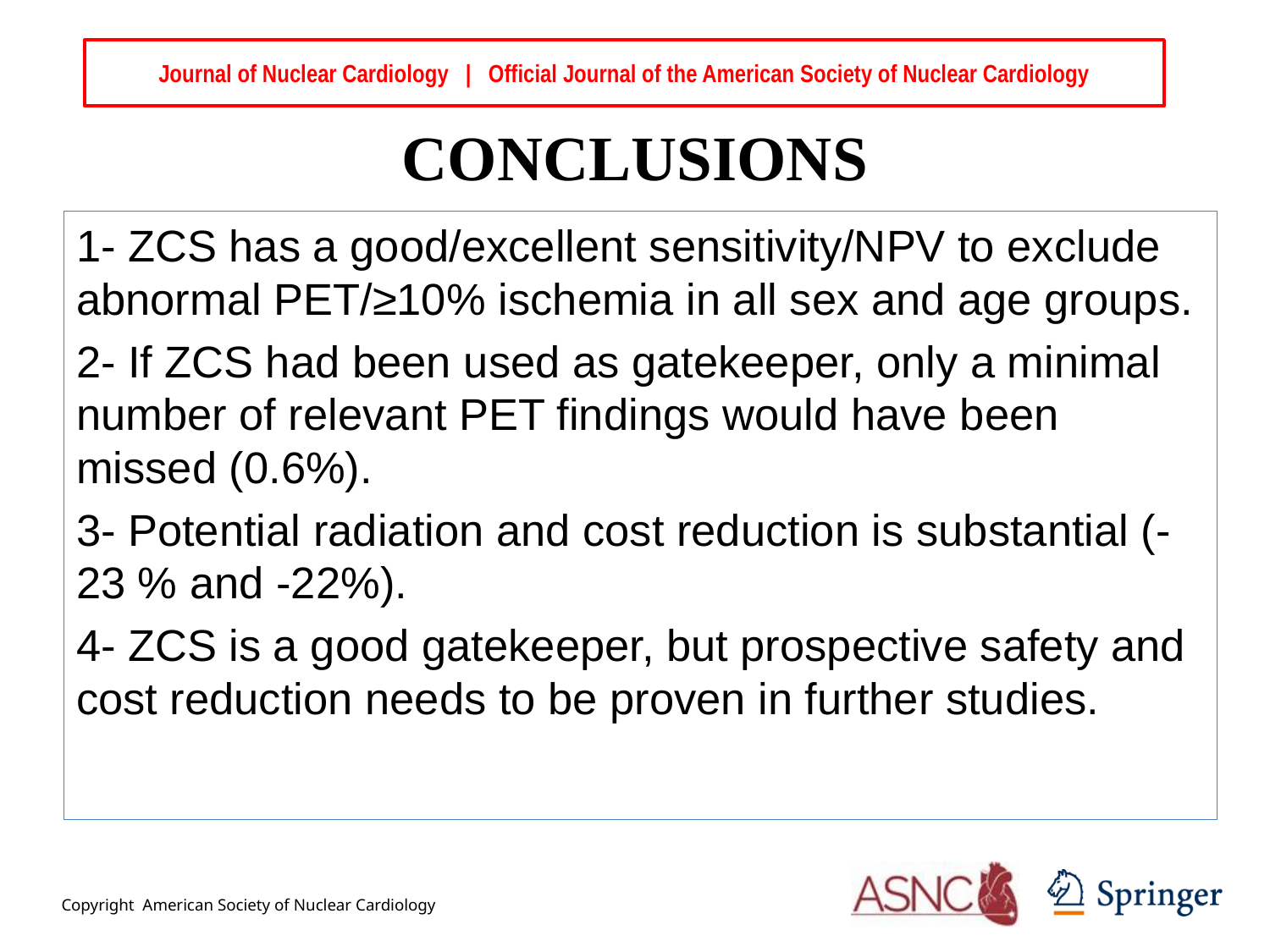

Journal of Nuclear Cardiology | Official Journal of the American Society of Nuclear Cardiology
# CONCLUSIONS
1- ZCS has a good/excellent sensitivity/NPV to exclude abnormal PET/≥10% ischemia in all sex and age groups.
2- If ZCS had been used as gatekeeper, only a minimal number of relevant PET findings would have been missed (0.6%).
3- Potential radiation and cost reduction is substantial (-23 % and -22%).
4- ZCS is a good gatekeeper, but prospective safety and cost reduction needs to be proven in further studies.
Copyright American Society of Nuclear Cardiology
